# Fusarium Head Blight and Rust Diseases in Soft Red Winter Wheat in the Southeast United States: State of the Art, Challenges and Future Perspective for Breeding

**DOI:** 10.3389/fpls.2020.01080

**Published:** 2020-07-16

**Authors:** Bikash Ghimire, Suraj Sapkota, Bochra A. Bahri, Alfredo D. Martinez-Espinoza, James W. Buck, Mohamed Mergoum

**Affiliations:** ^1^Department of Plant Pathology, University of Georgia, Griffin Campus, Griffin, GA, United States; ^2^Institute of Plant Breeding, Genetics, and Genomics, University of Georgia, Griffin Campus, Griffin, GA, United States; ^3^Department of Crop and Soil Sciences, University of Georgia, Griffin Campus, Griffin, GA, United States

**Keywords:** fusarium head blight, leaf rust, stripe rust, quantitative trait locus mapping, marker-assisted selection, resistance breeding, soft red winter wheat, southeast US

## Abstract

Among the biotic constraints to wheat (*Triticum aestivum* L.) production, fusarium head blight (FHB), caused by *Fusarium graminearum*, leaf rust (LR), caused by *Puccinia triticina*, and stripe rust (SR) caused by *Puccinia striiformis* are problematic fungal diseases worldwide. Each can significantly reduce grain yield while FHB causes additional food and feed safety concerns due to mycotoxin contamination of grain. Genetic resistance is the most effective and sustainable approach for managing wheat diseases. In the past 20 years, over 500 quantitative trait loci (QTLs) conferring small to moderate effects for the different FHB resistance types have been reported in wheat. Similarly, 79 *Lr*-genes and more than 200 QTLs and 82 *Yr*-genes and 140 QTLs have been reported for seedling and adult plant LR and SR resistance, respectively. Most QTLs conferring rust resistance are race-specific generally conforming to a classical gene-for-gene interaction while resistance to FHB exhibits complex polygenic inheritance with several genetic loci contributing to one resistance type. Identification and deployment of additional genes/QTLs associated with FHB and rust resistance can expedite wheat breeding through marker-assisted and/or genomic selection to combine small-effect QTL in the gene pool. LR disease has been present in the southeast United States for decades while SR and FHB have become increasingly problematic in the past 20 years, with FHB arguably due to increased corn acreage in the region. Currently, QTLs on chromosome *1B* from Jamestown, *1A, 1B, 2A, 2B, 2D, 4A, 5A*, and *6A* from W14, Ning7840, Ernie, Bess, Massey, NC-Neuse, and Truman, and *3B* (*Fhb1)* from Sumai 3 for FHB resistance, *Lr9*, *Lr10*, *Lr18*, *Lr24*, *Lr37, LrA2K*, and *Lr2K38* genes for LR resistance, and *Yr17 and YrR61* for SR resistance have been extensively deployed in southeast wheat breeding programs. This review aims to disclose the current status of FHB, LR, and SR diseases, summarize the genetics of resistance and breeding efforts for the deployment of FHB and rust resistance QTL on soft red winter wheat cultivars, and present breeding strategies to achieve sustainable management of these diseases in the southeast US.

## Introduction

Wheat (*Triticum aestivum* L.) is a staple food crop grown in 17% of the total world cropping area and contributes towards 18.3% of the global human calorie intake just next to rice (Peng et al., 2011; FAOSTAT, 2018). The United States (US) shares 7% of total global wheat production, and the crop ranks third among US field crops in terms of planted area, production, and gross farm income after corn and soybeans (USDA, 2020). Among the five major wheat classes in the US, soft red winter wheat (SRWW) common to the southeast US shares 15–20% of total area and 17% of total production (Vocke and Ali, 2013). However, this major cereal crop is under continuous threat due to several biotic and abiotic constraints resulting in a significant reduction in grain yield and quality (Limbalkar et al., 2018; Ghimire et al., 2020). Savary et al. (2019) reported 31 pests responsible for an estimated 21.5% economic yield loss in wheat. Among these, fusarium head blight (FHB), leaf rust (LR), and stripe rust (SR) are reported as the most problematic fungal diseases both in the southeast US and throughout the world (Chen et al., 2002; Cowger and Sutton, 2005; Milus et al., 2006; Kolmer et al., 2007; Walter et al., 2016; Kolmer et al., 2019). SRWW production in the southeast has decreased by nearly 61% over the past decade (2008 to 2019) due to several factors of which FHB, LR, and SR are the most prominent (https://usda.library.cornell.edu/concern/publications/5t34sj573). While FHB alone was ranked the second most challenging disease in the US midwest, Canada, South Brazil, Paraguay, Uruguay, and Argentina next to tan spot, both FHB and LR were ranked the topmost diseases in China and across the globe (Savary et al., 2019).

FHB, also widely known as wheat scab, head scab or scab, is a floral disease of wheat caused by *Fusarium graminearum* Schwabe [teleomorph *Gibberella zeae* (Schwein.) Petch]. FHB is of great concern to wheat producers because of its yield-reducing ability as well as food and feed safety threat associated with harmful mycotoxin contamination mostly by deoxynivalenol (DON) in the infected grain (McMullen et al., 1997; Dweba et al., 2017; ElDoliefy et al., 2020). Smith (1884) first described the disease symptoms and morphological characters of *Fusisporium culmorum* (present day *Fusarium culmorum*) causing FHB, and epidemics were also reported from the UK in 1884 (Hao et al., 2020). FHB was reported in the US states of Indiana, Delaware, and Ohio in the 1890s (Chester, 1890; Arthur, 1891). One hundred years later, a major FHB epidemic affected over 10 million acres of wheat in Minnesota, North Dakota, South Dakota, and the Canadian province of Manitoba causing over $1 billion in yield losses in 1993 alone and $7.7 billion gross loss over 1993–2001 (McMullen et al., 1997; Nganje et al., 2004).

FHB has become increasingly problematic in the past 20 years in the southeast US possibly due to corn acreage expansion. Increased corn acreage in the US (6 million hectares more in 2018) including all southeastern states since 1990 along with diminishing wheat acreage (about 12 million hectares less in 2018) also correlates with increased FHB epidemics in the region (USDA, 1990; USDA, 2018; https://www.ers.usda.gov/data-products/chart-gallery/gallery/chart-detail/?chartId=76955). Cowger and Sutton (2005) reported a total of $13.6 million yield loss from five southeastern states due to FHB in 2003. Nearly 50% disease incidence was observed in experimental plots in 2013–2014, while incidence as high as 80% was observed in some commercial wheat fields across Georgia in 2014–2015 and 2018–2019 (Lilleboe, 2014; Buck and Youmans, 2015). As a result, wheat millers have incurred higher costs for mycotoxin testing, additional cleaning and blending, as well as additional shipping costs to access better quality grain.

The ability of the FHB pathogens to produce mycotoxins poses a persistent global threat to human and livestock health. The US Food and Drug Administration (FDA) has restricted DON levels below 1 ppm for finished wheat products such as flour and bran consumed by human and 5–10 ppm for all livestock feed (FDA, 2010). Type-B trichothecenes, such as DON, are acutely phytotoxic and can act as virulence factors on a sensitive host maintaining positive relationship with FHB severity as observed across several studies (O’Donnell et al., 2000; Lemmens et al., 2005; Buerstmayr et al., 2019). Nevertheless, Cowger and Arellano (2010) observed that asymptomatic wheat field with low infected grain might also constitute higher DON due to late infection and rainfall immediately after anthesis. Recently, there have been increased concerns about higher mycotoxin levels in wheat straw for livestock feed due to reduced DON levels observed in grain during grain fill compared to non-grain tissues such as rachises and glumes (Cowger and Arellano, 2013; Bissonnette et al., 2018).

LR and SR, also known as brown rust and yellow rust, caused by *Puccinia triticina* Eriks. *(Pt)* and *Puccinia striiformis* Westend. *(Ps)* respectively, are the most common rust diseases of wheat; the other being stem rust caused by *Puccinia graminis* subsp. *graminis* Pers.:Pers (Kolmer, 2005; Bolton et al., 2008). These widespread and destructive foliar diseases have been closely scrutinized due to the continuous evolution of the novel and more virulent pathogen races which are difficult to manage (Kolmer, 2005; Kolmer et al., 2007). Reports on physiologic races of LR from the former Soviet Union and the US originated as early as 1960 with 5–100% disease severity observed in the field (Herr, 1961). Huerta-Espino et al. (2011) reported an estimated 3 million tons of wheat yield loss worth of $350 million in the US due to LR between 2000 and 2004. LR normally reduces grain yield by 5–15%; however, losses up to 40% have been reported depending on the climatic conditions, time and duration of infection, and resistance levels of wheat cultivars (Samborski, 1985; Marasas et al., 2004; Kolmer et al., 2007). Earlier reports on describing phenotypes of *Pt* as well as yield loss assessment in SRWW from the south Atlantic States also reflect the importance of LR in this region (Kolmer, 2002; Green et al., 2014). SR is the most frequent disease of wheat in the western US causing up to 70% of yield loss (Chen et al., 2002). Since 2000, it has been widespread in the southeast US and become destructive on SRWW with the emergence of new races (Chen et al., 2002; Milus et al., 2006). Interestingly, Khan et al. (1997) revealed that for every 1% increase in rust severity, there is a 1% reduction in the wheat grain yield. A photosynthetically active wheat flag leaf significantly impacts grain formation, therefore higher yield reduction together with decreased number of kernels per spike, lower kernel weight, plant biomass, and harvest index often occur with an increased infection on flag leaves (Chester, 1946; Bolton et al., 2008; Huerta-Espino et al., 2011; Green et al., 2014).

Reviews at the turn of the twenty-first century included epidemiology (Parry et al., 1995) and conventional breeding of FHB (Mesterhazy, 1995; Miedaner, 1997; Mesterházy et al., 1999). More recently, reviews of the literature have been published on diverse fields of FHB resistance including QTL mapping and marker-assisted selection (MAS) (Buerstmayr et al., 2009; Gupta et al., 2010; Shah et al., 2017), genomic selection (GS) (Larkin et al., 2019), and resistance breeding (Kosová et al., 2009; Buerstmayr et al., 2014; Steiner et al., 2017; Buerstmayr et al., 2019; Zhu et al., 2019). Similarly, review papers on broad aspects of breeding and genomic selection (Todorovska et al., 2009), epidemiology (Eversmeyer and Kramer, 2000; Brown and Hovmøller, 2002; Chen, 2005; Bolton et al., 2008; Jin et al., 2010; Zhao et al., 2016), and host resistance (Kolmer, 1996; Chen, 2007; Kolmer et al., 2007; Singh et al., 2011; Kolmer, 2013; Ellis et al., 2014; Aktar-Uz-Zaman et al., 2017; McIntosh et al., 2017) on LR and SR have been presented. In addition, several QTL meta-analyses on FHB (Liu et al., 2009; Löffler et al., 2009; Venske et al., 2019), LR (Soriano and Royo, 2015), and SR (Cheng et al., 2018) have added additional evidence to the breeding efforts undertaken globally. However, to our knowledge, there is no single review focusing on SRWW breeding for resistance to FHB, LR, and SR in the southeast US.

## Epidemiology and Management of FHB

*F. graminearum* is a homothallic, facultative parasite of wheat that normally overwinters as a saprophyte in the plant debris of small grains and corn which serves as a reservoir for primary inoculum the next season (McMullen et al., 2012; Dweba et al., 2017). The pathogen has a brief biotrophic phase until it colonizes the living tissue to maintain the necrotrophic relation with its host (Goswami and Kistler, 2004). However, it is still unclear whether *F. graminearum* is a true hemibiotroph or not (Dweba et al., 2017). The fungus has a wide host range and can infect several hosts including wheat, barley, rice, oats and causes *Gibberella* stalk and ear rot disease on corn (Goswami and Kistler, 2004; McMullen et al., 2012). The FHB pathogens can also cause seedling blight, root and stem rot if infected wheat seeds are planted the following year.

**Figure 1:**
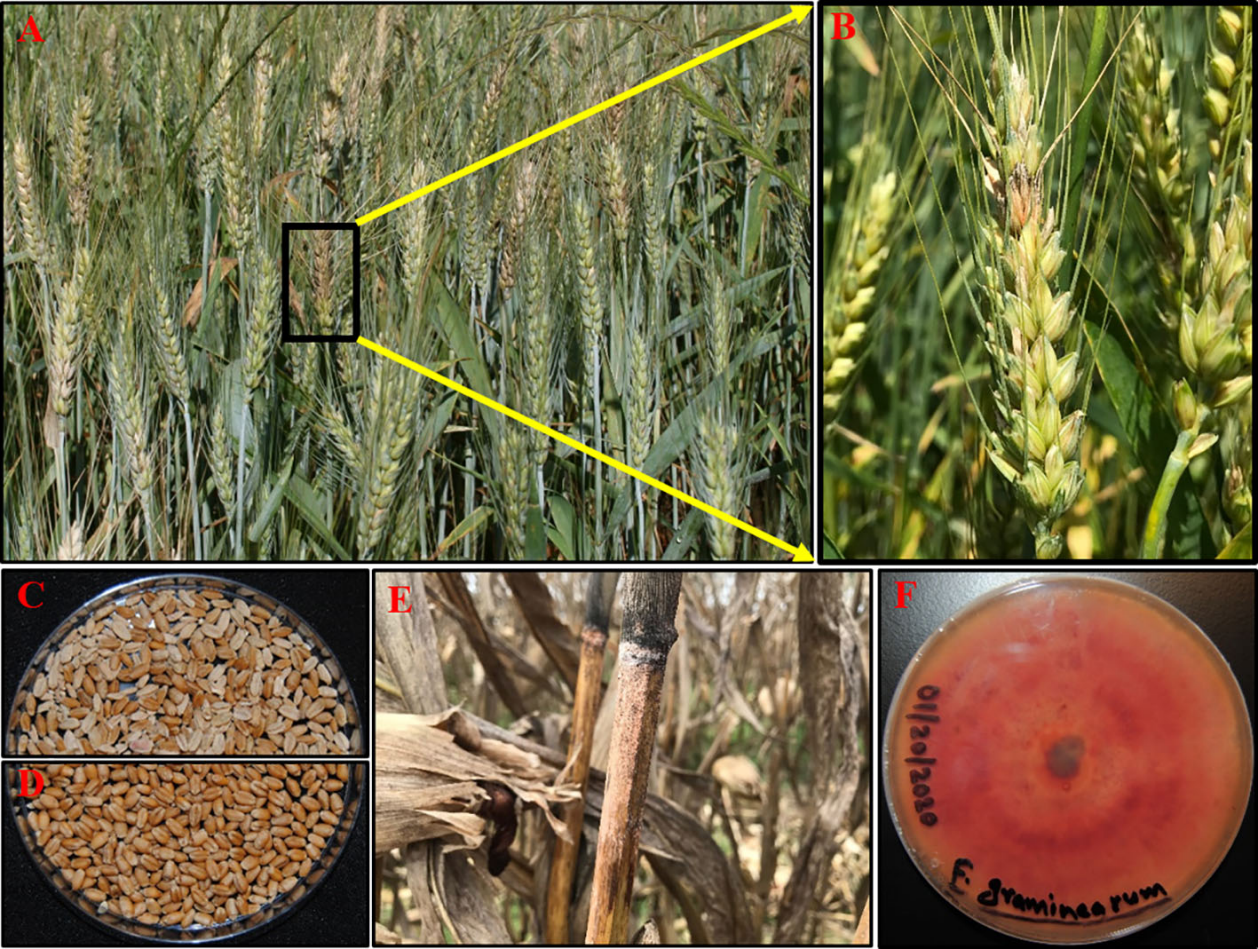
Fusarium head blight (FHB) symptom; FHB disease in the field used to assess FHB incidence (type I resistance) **(A)**, infected spike with orange-colored sporodochia used to assess FHB severity (type II resistance) **(B)**, infected seed used to assess *Fusarium*-damaged kernel (FDK) (type IV resistance) **(C)** compared to healthy seed lot **(D)**, *F. graminearum* overwintering in corn stalk **(E)**, and FHB isolate cultured in potato dextrose agar (PDA) plate showing characteristic pinkish-red colony **(F)**. (Source: **Figures 1A**–**F**, B. Ghimire).

A warm and moist environment with high relative humidity (above 90%) can stimulate ascospore and conidial dispersal by wind, rain or insects to healthy wheat spikes to trigger infections during anthesis (McMullen et al., 1997; Schmale III et al., 2006). Cowger et al. (2009) revealed that post-anthesis moisture is crucial for disease development and contributes to increased FHB severity and DON contamination levels. The fungus colonizes wheat anthers and eventually the middle rachis blocking the vascular cambium; thus the portion of the wheat head above the affected area stops growing and exhibits a typical premature bleaching symptom (**Figures 1A, B**) (Pritsch et al., 2000; Buerstmayr and Buerstmayr, 2016). The most discernable symptoms later in the season are the formation of pinkish-orange colored asexual fruiting structures (sporodochia) in the spikelet hosting both microconidia and macroconidia (**Figure 1B**) (Schmale III et al., 2006; McMullen et al., 2012). Infected wheat spikes often harbor shriveled tombstone grains that are discolored with low kernel weight and inferior quality due to high mycotoxin contamination (**Figure 1C**). It is also common to observe black-colored overwintering perithecia and sporodochia on matured wheat spikes as well as corn debris (**Figure 1E**) which serve as primary inoculum for next season continuing the disease cycle (McMullen et al., 2012). The pathogens can be easily isolated and cultured on the growth media from infected wheat spikes or corn debris allowing morphological and genetic analyses (**Figure 1F**).

The filamentous, ascomycetous fungus *F. graminearum* was perceived as a single panmictic species responsible for FHB until the end of the 20^th^ century (O’Donnell et al., 2000). At present, *F. graminearum* producing the 15ADON chemotype is considered one of the 16 phylogenetically distinct species within the *F. graminearum* species complex (FGSC) primarily responsible for FHB in the US including the southeast region (Sarver et al., 2011; Bec et al., 2015; Ghimire et al., 2019a; Ghimire et al., 2019b). There has been a gradual shift from 15ADON to more toxigenic 3ADON population in the US and Canada presumably due to climate change and fitness advantages towards a more aggressive chemotype with exact reason not known yet (Ward et al., 2008; Puri and Zhong, 2010; Schmale et al., 2011). Additionally, the high proportion of recently evolved *NX-2* chemotype in the northern US and Canada foretells the possible threat of this novel strain in the southeast US (Kelly and Ward, 2018; Lofgren et al., 2018).

Management of FHB requires an integrated approach incorporating the use of cultural practices, fungicides, and host resistance. Since planting of wheat crop directly on corn stubble is a common practice, interrupting the corn–wheat cropping pattern and adopting tillage practices in no-till cropping systems are the best viable cultural practices (McMullen et al., 1997; McMullen et al., 2012). The adoption of proper crop rotation along with the removal of infected residue could reduce FHB infection and DON by 30% (McMullen et al., 2012). The use of demethylation inhibitor (DMI) fungicides in combination with a moderately resistant cultivar at anthesis was partially successful in managing FHB and DON level (McMullen et al., 2012; Madden et al., 2014; Martinez-Espinoza et al., 2014; Bowen et al., 2016; Paul et al., 2019); however, the effective use of fungicides within the short window of anthesis is challenging *per se* (Cowger et al., 2016).

The development of FHB resistant wheat cultivars is the single most cost-effective and sustainable approach to manage this disease. Although complete FHB resistance has not been obtained yet (Steiner et al., 2017), identification and validation of major and minor effect QTLs and their introgression through diverse breeding methodologies including recently implemented methods such as marker-assisted and genomic selections are of paramount important. These methods have allowed gene pyramiding for durable resistance across wheat breeding programs (Buerstmayr et al., 2009; Li et al., 2016b; Shah et al., 2017; Zhao et al., 2018; Zhu et al., 2019). Meanwhile, the catastrophic epidemics in the 1990s resulted in the initiation of the US wheat and barley scab initiative (USWBSI; https://scabusa.org/) in 1997 (McMullen et al., 2012). The initiative has used a multi-state, integrated approach to minimize FHB risk through breeding for FHB resistance sharing germplasm through uniform scab nurseries, weather-based forecasting systems (FHB Alerts and ScabSmart), and uniform fungicide trials (McMullen et al., 1997). In the southeast US, SunGrains (http://www.sungrains.lsu.edu/about.shtml) was established in 2005 with currently seven universities collaborating to develop superior wheat germplasm with insect and disease resistance by sharing and screening germplasm.

## Epidemiology and Management of LR and SR

Unlike the FHB pathogen, *Pt* and *Ps* which cause LR and SR in wheat, respectively, are obligate parasites and cannot be cultured outside the host tissue (Zhao et al., 2016). These biotrophic fungal pathogens interact with host resistance genes in a gene-for-gene manner (Chen, 2007; Kolmer, 2013; Zhao et al., 2016). *Pt* and *Ps* are heteroecious and require two genetically unrelated hosts to complete their life cycle (Roelfs et al., 1992; Bolton et al., 2008; Jin et al., 2010; Zhao et al., 2013): the primary host wheat in which it completes its asexual life cycle and alternate hosts, *Thalictrum speciosissimum* (meadow rue) for LR and *Berberis* spp. for SR, in which they complete the sexual life cycle. Rust pathogens are macrocyclic and have five different spore stages *i.e.*, teliospores, basidiospores, urediniospores, pycniospores, and aeciospore (Bolton et al., 2008; Jin et al., 2010; Kolmer, 2013). LR and SR are polycyclic diseases; urediniospores can re-infect wheat under conducive climatic conditions leading to disease epidemics (Bolton et al., 2008; Kolmer, 2013; Chen et al., 2014). At later growing stages as temperatures rise, thick-walled black telia are formed which enable the pathogens to survive non-host periods (Chen et al., 2014). With favorable environmental conditions, the haploid basidiospores formed from the telia are wind-dispersed to nearby alternate hosts to form pycnium which later forms aeciospores to infect the primary host thus completing the life cycle (Bolton et al., 2008; Kolmer, 2013; Chen et al., 2014).

**Figure 2:**
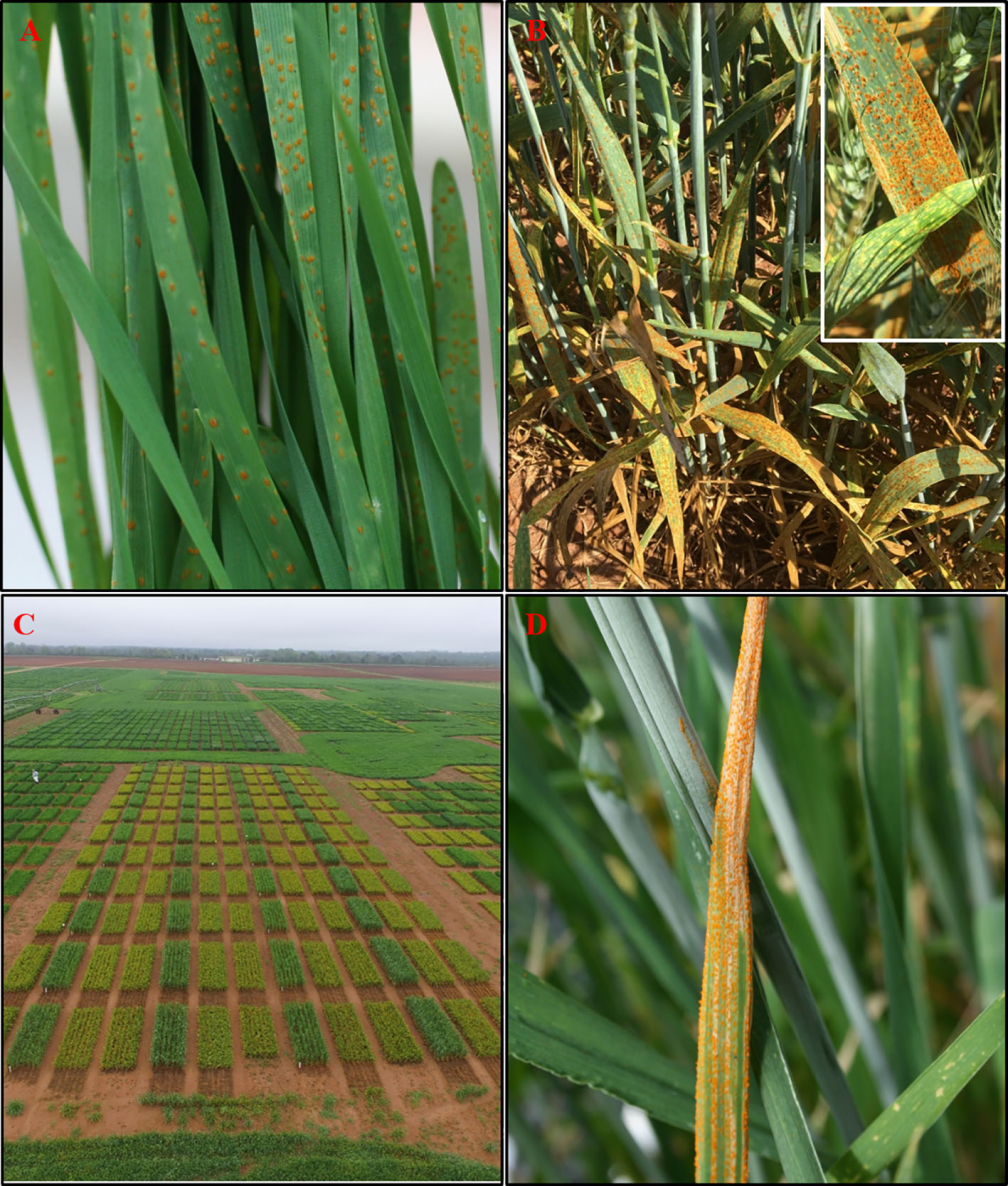
Wheat rusts; leaf rust (LR) uredinia on wheat seedlings in greenhouse **(A)**, and adult plants in the field **(B)** used to assess seedling and adult plant resistance, respectively, stripe rust (SR) disease in the field plot compared to healthy wheat **(C)**, and SR uredinia showing the elongated stripes typical of the disease on the flag leaf of adult plants in the field **(B)**. Field photos are taken from southwest Georgia research and education center, Plains, GA (Source: **Figures 2A, B**, S. Sapkota; **2C, D**, A. D. Martinez-Espinoza).

The typical symptoms of LR on wheat leaves appear as round lesions with orange-brown urediniospores on the upper leaf surface (**Figures 2A, B**) (Roelfs et al., 1992; Zhao et al., 2016). SR symptoms appear as yellow to orange uredinial pustules arranged in stripes usually between veins on leaves, glumes, and awns (**Figure 2D**) (Chen et al., 2014). Usually, LR and SR appear successively in the field during warm and dry, and cool and humid periods, respectively. *Pt* and *Ps* can infect wheat leaves if exposed to 4–8 hours of dew period with a temperature of 10–25°C and 5–12°C, respectively (Roelfs et al., 1992; de Vallavieille-Pope et al., 1995). However, new races of SR adapted to high temperature became prevalent worldwide, leading to epidemics in warmer areas where the disease was not problematic (Milus et al., 2008; Mboup et al., 2009; Hovmøller et al., 2010). The occurrence of several identical multilocus genotypes has justified that wheat rust spores can undergo long-distance dispersal of thousands of miles across continents and ocean by wind and intercontinental exchanges (Brown and Hovmøller, 2002; Kolmer, 2005; Bahri et al., 2009; Hovmøller et al., 2010; Walter et al., 2016; Kolmer et al., 2019).

The populations of rust pathogens in the US have been dynamic with the discovery of new rust resistance genes, migration of virulence phenotypes, clonal reproduction, and mutation of rust urediniospores as documented by annual surveys conducted since the 1930s for LR (Mains and Jackson, 1926; Ordoñez and Kolmer, 2009; Kolmer, 2019) and the 1960s for SR (Chen et al., 2002; Line, 2002). Since Johnston (1961) reported a total of 16 physiologic races of LR from 22 US states, new races have appeared while others have disappeared. A collection of rust isolates from Georgia, North Carolina, South Carolina, and Virginia in 1999 revealed that virulence phenotype MBRK (virulence to LR genes *Lr1, Lr3, Lr3ka, Lr11, Lr30, Lr10, Lr14a*, and *Lr18*) at 38.7% and TLGF (virulence to *Lr1, Lr2a, Lr2c, Lr3, Lr9, Lr11, Lr14a*, and *Lr18*) at 33.8% of isolates were the most common phenotypes suggesting the south Atlantic states were a single epidemiological area for LR (Kolmer, 2002). However, a recent study updated the presence of 40 multilocus genotype groups widely distributed across wheat growing regions worldwide of which six exist in North America (Kolmer et al., 2019). MBTNB and MCTNB phenotypes with virulence to *Lr11* and *Lr18* are typical to the southeastern states and Ohio valley region. Similarly, Wan and Chen (2014) identified 146 races of stripe rust in 2010 compared to 21 races in 2000 in the US (Chen et al., 2002). Prior to 2009, SR races with virulence against *Yr1, Yr3, Yr8, Yr9, Yr10, Yr11, Yr12, Yr16, Yr17, Yr18, Yr19*, and *Yr20* were frequently reported in the southeast US; whereas, *Yr6, Yr7, Yr8, Yr9, Yr27, Yr43, Yr44*, and *YrExp2* virulence combinations have been identified in the last few years (https://striperust.wsu.edu/races/data/).

Similar to the management of FHB, an integrated management approach is the most effective means of controlling LR and SR (Roelfs et al., 1992). Cultural practices such as eradication of volunteer plants, crop debris, and alternate hosts (if present) can reduce primary inoculum (Roelfs et al., 1992) but do not guarantee complete freedom from rust spores in the field since urediniospores are carried long-distance by wind. Aerial dispersal of rust spores makes quarantine methods ineffective to control this disease. Although an effective management of rust diseases is possible by the use of fungicides, the approach is not always economically feasible (Bowen et al., 1991; Roelfs et al., 1992; Buck et al., 2009; Buck et al., 2011). Genetic resistance is therefore the preferred method to manage rust diseases. To date, 79 *Lr* genes and more than 200 QTLs, as well as 82 *Yr* genes and 140 QTLs have been identified for both seedling and adult plant LR and SR resistance, respectively (Rosewarne et al., 2013; McIntosh et al., 2017; Sapkota et al., 2019b).

## Genetics of FHB Resistance and Breeding Strategies

### Types of FHB Resistance

**Figure 3:**
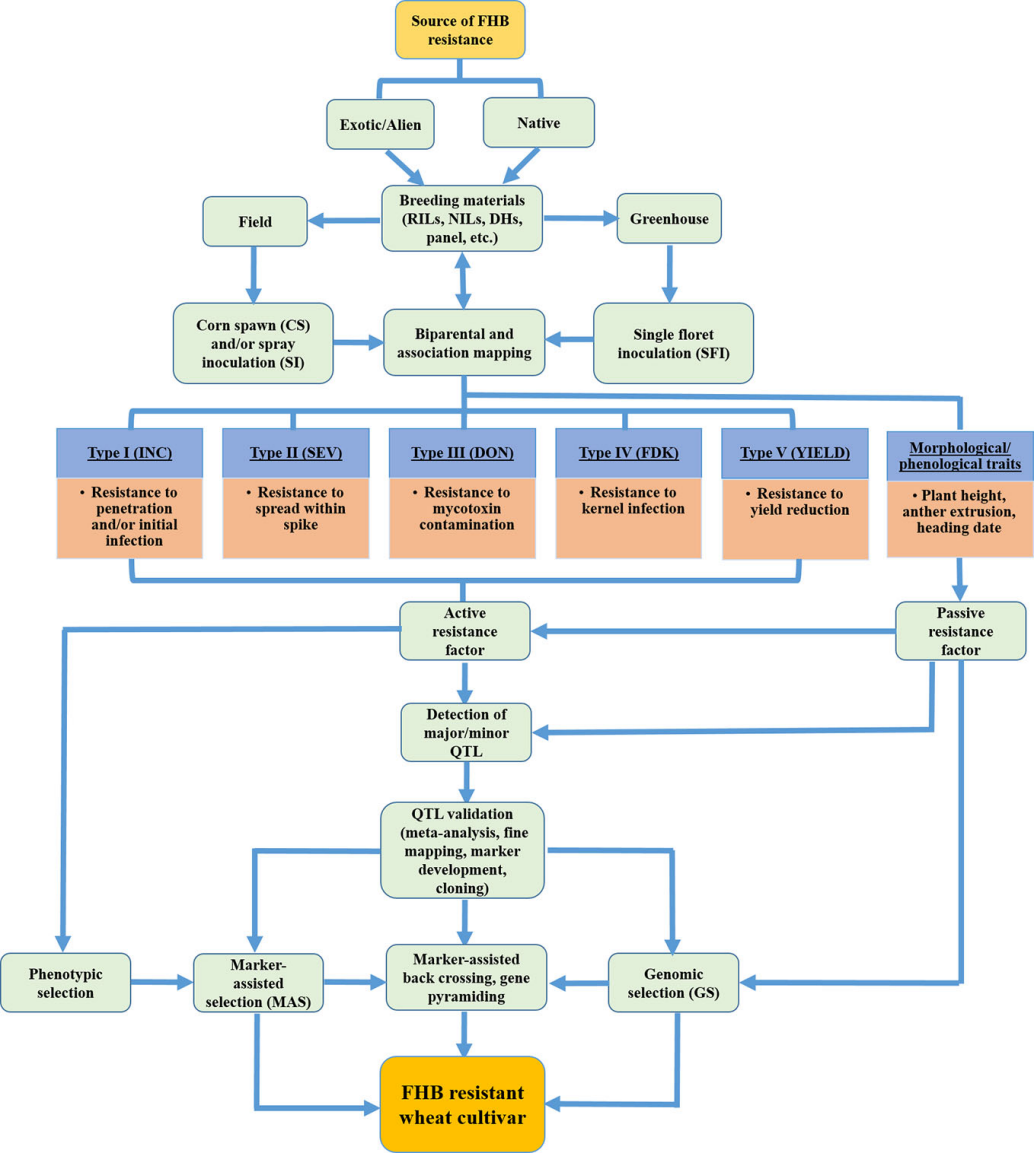
Schematic flow diagram showing overall breeding effort for assessing FHB resistance types, QTL detection and validation, and development of FHB resistant wheat cultivars. (Source: **Figure 3**, B. Ghimire).

Host plant resistance to FHB is “horizontal” and neither species nor race-specific in nature (Mesterházy et al., 1999); rather resistance has low heritability, is quantitatively inherited and polygenic trait. There are no reports of genetic immunity to FHB; however multiple resistance genes can overlap to confer one or the other types of resistance (Parry et al., 1995; Ma et al., 2020). Type I and type II resistance was presented initially by Schroeder and Christensen (1963). This was later broadened to the five different types of host resistance mechanisms to FHB widely accepted to date (Mesterhazy, 1995; Mesterházy et al., 1999). Type I (FHB incidence) involves resistance to penetration and/or initial infection; type II (disease severity) is the condition when host plants resist spread of infection from one spikelet to another within an infected spike; type III reduces accumulation of mycotoxin(s) in the infected kernel; type IV provides resistance to kernel infection also known as *Fusarium*-damaged kernel (FDK); and type V provides resistance to yield loss (**Figure 3**). FHB resistant cultivars typically exhibit more than one resistance type since each QTL/gene can produce several resistance reactions (Dweba et al., 2017). Although type II resistance has been widely studied as the primary resistance type and type V is almost a neglected class, studies on the sources of type III and type IV resistance are becoming of primary interest for most of the wheat breeding programs due to increased health concerns associated with harmful vomitoxins (Venske et al., 2019; Zhu et al., 2019).

### Sources of FHB Resistance

There are two major approaches for introducing FHB resistance in SRWW; deployment of resistant QTL from alien and exotic gene pools such as *Fhb1* from Sumai 3 and exploitation of native sources of resistance from local cultivars (Brown-Guedira et al., 2008; Steiner et al., 2017). Despite the complexity of FHB resistance, the larger genetic variation from both exotic and native sources has been harnessed for deploying several resistance traits in modern wheat cultivars.

#### Exotic and Alien Sources of FHB Resistance

Sumai 3 is a highly resistant spring wheat cultivar developed from a cross between resistant parents Funo and Taiwan Xiaomai and was released by the Suzhou Institute of Agricultural Sciences in 1970 (Cuthbert et al., 2006; Buerstmayr et al., 2009). Sumai 3 is the most widely used donor parent to deploy the major effect QTL *Fhb1* in several wheat breeding programs throughout the world (Zhu et al., 2019). While Sumai 3 has been extensively used in North America, other regions are either relying on different sources of *Fhb1* such as Shinchunaga in Japan and Norin 129 and Nigmai 9 in China or even dropping *Fhb1* due to preferential selection for the stem rust gene *Sr2* in International Maize and Wheat Improvement Center (CIMMYT) as both of these genes are linked in repulsion (Buerstmayr et al., 2019). Besides Sumai 3, novel sources of FHB resistance from several other Asian cultivars or landraces such as Chokwang conferring type II resistance from Korea and Wangshuibai, Nyu-Bai and Nobeokabozu and their descendants CM-82036 and Ning7840 from China are widely used in US wheat breeding programs (Yang et al., 2005; Buerstmayr et al., 2009; Buerstmayr et al., 2014; Li et al., 2016b; Buerstmayr et al., 2019). Before the introduction of Sumai 3, US wheat breeding programs primarily used type I resistance sourced from Brazilian donor cultivar Frontana with stable QTL reported on chromosomes *3AL* and *5A* (Steiner et al., 2004; Buerstmayr et al., 2009). In addition, wheat relatives including wild emmer (*T. dicoccoides*) were also successfully used in hard red spring wheat (HRSW) to release several adapted cultivars including Steele-ND (Mergoum et al., 2005). Nevertheless, the occurrence of *Fhb2* and *Fhb5* QTLs in Sumai 3 in addition to *Fhb1* has made this spring wheat cultivar a universal donor in FHB resistance breeding (Buerstmayr et al., 2019).

McVey is considered the first Sumai 3-derived US cultivar and was developed in Minnesota in 1999 (Hao et al., 2020). Since then, several top derivatives of Sumai 3 have been developed in the US by University programs: Alsen (2002–2006) (Frohberg et al., 2006), Glenn (2007–2011) (Mergoum et al., 2006), Barlow (2012–2015) (Mergoum et al., 2011), SY Soren (2016), and SY Ingmar (2017–2018) in North Dakota, Faller (2009–2012) (Mergoum et al., 2008) and Prosper (2013–2015) (Mergoum et al., 2013) in Minnesota, Prevail (2016–2017) in South Dakota, and AAC Brandon (2016–2018) in Manitoba, Canada (Zhu et al., 2019; Hao et al., 2020). Private seed companies are also actively involved in developing FHB resistant SRWW. For example, Pioneer Breeding Company developed 25R18 (1999), 25R42 (2001), 25R35 (2003), 25R54 (2003), INW0412 (2004), and 25R51 (2005) exploiting both Asian (*3BS* and *5AS*) and/or European (*1B* and *3A*) sources in the native background (Griffey, 2005; Zhu et al., 2019). Though more than 20 HRSW cultivars with Sumai 3 in their pedigrees are widely grown in the northern US and Canada, a negligible number of SRWW cultivars have been released for the southeast US (Steiner et al., 2017; Zhu et al., 2019).

The *Fhb1* locus previously reported as *Qfhs.ndsu-3BS* was mapped as a single Mendelian gene in a high-resolution mapping population (Cuthbert et al., 2006). Bakhsh et al. (2013) suggested *Fhb1* does not have any deleterious effects on agronomic and end-use quality traits in hard red winter wheat (HRWW) and near-isogenic lines (NILs) with *Fhb1* locus reduced FHB disease severity and infected kernels by 23 and 27%, respectively (Pumphrey et al., 2007). Further, the unique ability of *Fhb1* to detoxify DON to DON-3-O-glucoside can be further explored to reveal the mechanism of detoxification for wider applications (Lemmens et al., 2005).

Oliver et al. (2005) conducted an extensive evaluation of several wheat-alien species derivatives (*Roegneria kamoji, R. ciliaris, Leymus racemosus, Thinopyrum ponticum, Th. elongatum, Th. junceum, Th. intermedium, Dasypyrum villosa, Secale cereal*, and *Avena sativa*) and concluded these were an invaluable gene pool and could serve as novel sources for FHB resistance. FHB resistance is also present in more closely related species including *Triticum tauschii* (Oliver et al., 2005), *T. macha* (Steed et al., 2005), *T. timopheevii* (Malihipour et al., 2017), and *T. spelta* (Góral et al., 2008), and distantly related species Agropyron (*Elymus spp*.) (Ban, 1997) and *Lophopyrum elongatum* (Shen and Ohm, 2006). *T. dicoccoides* and tall wheatgrass have been widely exploited in the southeast US donating the major FHB QTL *Qfhs.ndsu-3AS* and *Qfhs.pur-7EL*, respectively (Brown-Guedira et al., 2008).

#### Native Sources of FHB Resistance

Native sources of resistance are more convenient to use compared to exotic sources due to higher adaptability as well as the presence of good agronomic and post-harvest quality traits. After the identification of the first native source of FHB resistance in SRWW Freedom in 1991 with a QTL mapped to chromosome *2AS*, several other resistance sources were explored (Griffey, 2005). The most popular native cultivars documented with FHB resistance include: Massey (*3BL*) (1981), COKER 9474 (*3BS*), Ernie (*1A*/*2B/3BL/4A/4BL/5A/6A*) (1994), Foster (1995), Patton (*Fhb2/3B/6B*), Roane, Hondo, Heyne (*3AS/4DL/4AL*) (1998), Goldfield (*2B/7B*), Wesley (*Fhb1*) (1999), McCormick (*3BS/2D/5A*), Tribute (2002), NC-Neuse (*1A/4A/6A*), Truman (*2ASc/2DS/3DS*), INW0304 (*2B*), IL94-1653 (*2B/4B)* (2003), Cecil, INW0411 (*Fhb1/1B/3A*) (2004), Bess (*1A/2B/3B*), NY88046-8138, COKER 9511, WestBred X00-1079 (2005), and USG3555 (2007) (Van Sanford et al., 1997; Gilsinger et al., 2005; Griffey, 2005; Liu et al., 2007; Brown-Guedira et al., 2008; Griffey et al., 2008; Bonin and Kolb, 2009; Kang et al., 2011; Eckard et al., 2015; Islam et al., 2016; Bai et al., 2018; Murphy et al., 2019; Zhu et al., 2019). The SRWW cultivar Jamestown developed from the cross between Roane and Pioneer Brand 2691 by the Virginia Agricultural Experiment Station in 2007 is considered as a strong native source of FHB resistance (*1BL*) and is widely used a donor parent and FHB resistance check in the annual Southern Uniform Winter Wheat Scab Nursery (SUWWSN) (Griffey et al., 2010a; Zhu et al., 2019).

### QTL Mapping for FHB Resistance and Validation

With the rapid advancements in genotyping along with reduced costs, it has become a routine approach to detect QTL associated with disease resistance mostly using conventional biparental QTL mapping (Buerstmayr et al., 2019; ElDoliefy et al., 2020; Ma et al., 2020). Since the first QTL mapping studies conducted independently by Waldron et al. (1999) and Bai et al. (1999), almost every wheat breeding program has integrated QTL mapping for FHB resistance with other important agronomic and quality traits (**Figure 3**). In 2003, several research groups initiated MAS (Yang et al., 2003; Zhou et al., 2003) leading to the first MAS-developed wheat cultivar Sabin from the University of Minnesota Agricultural Experimental Station in 2009 (Anderson et al., 2012). To date, more than 500 QTLs conferring resistance to different FHB traits except for grain yield (type V resistance) have been reported (Buerstmayr et al., 2019; Venske et al., 2019). However, only 20% are major effect QTLs (Buerstmayr et al., 2019).

Although hundreds of QTLs have been identified from all 42 wheat chromosomes, few have only been validated and successfully deployed in breeding programs to date (Buerstmayr et al., 2009; Steiner et al., 2017; Buerstmayr et al., 2019; Venske et al., 2019). Several QTL meta-analyses performed for FHB resistance in wheat at different time periods have also cross-validated to ensure the applicability of promising QTL (Liu et al., 2009; Löffler et al., 2009; Mao et al., 2010; Venske et al., 2019). For example, a comprehensive QTL meta-analysis conducted by Venske et al. (2019) utilized 556 QTLs from multiple projects and concluded that only two out of ten FHB-responsive genes recovered from meta-QTL 1/chr. *3B* encoding for glycosiltransferase and cytochrome P450 were validated while the rest of the promising loci required further study. The analysis also revealed that the wheat B sub-genome had the largest number of QTL (238 QTLs) mapped further down on the chromosome 3B region (81 QTLs). The highest number of QTLs (41.5%) was reported for type II resistance while the fewest QTLs were for type I resistance (11.5%). The meta-analysis emphasized that future breeding activities should be directed towards fine mapping for QTL validation and the development of diagnostic markers for routine use in MAS and GS.

However, it is often questionable whether markers can be validated in a diverse background to enhance the visibility of the QTL mapping results. Nonetheless, Li et al. (2016a) observed very few markers (~3%) holding reproducible marker-trait association while testing 364 genome-wide informative SSR and sequence-tagged site (STS) markers associated with type II FHB resistance. Additionally, Petersen et al. (2017) found that only one QTL (*Qfhb.nc-2B.1*) and its associated marker contributed resistance to disease severity and FDK and was identified as the best candidate for use in MAS among twelve genomic loci identified from diverse wheat nurseries. The same research group previously revealed that Kompetitive Allele-Specific PCR (KASP) markers, *Qfhb.nc-1A, Qfhb.nc-1B*, and *Qfhb.nc-6A* developed from SRWW NC-Neuse are good candidates for use in MAS (Petersen et al., 2016). With the identification of the *Fhb1* locus from Sumai 3 in 1999, several diagnostic markers have been developed allowing the successful introgression of this durable resistance locus in SRWW through MAS (Cuthbert et al., 2006; Bernardo et al., 2012; Steiner et al., 2017; Su et al., 2018).

FHB resistance is partially additive, so gene pyramiding is feasible using marker-assisted backcrossing or using either a single or combination of different selection strategies including phenotypic selection, MAS, and GS (**Figure 3**) (Shah et al., 2017). Agostinelli et al. (2012) suggested that an initial round of phenotypic selection for FHB traits at a moderate intensity will increase the homozygous resistant alleles at the major locus thus intensifying the MAS in the next round. Zhou et al. (2003) suggested MAS for major QTL during the seedling stage followed by phenotypic selection after anthesis benefited from effective FHB screening. Gupta et al. (2010) reviewed the progress and prospects of several aspects of MAS strategies such as AB-QTL, mapping-as-you-go, marker-assisted recurrent selection, and genome-wide selection. To date, *Fhb1*, *Fhb2*, and *Qfhs.ifa-5A* derived from Sumai 3 are reported the most used/validated and therefore, pyramided type II resistance QTL (Cuthbert et al., 2006; Cuthbert et al., 2007; Xue et al., 2011; Balut et al., 2013; Steiner et al., 2017; Su et al., 2018). Several authors have confirmed an enhanced type III resistance when *Fhb1* locus was pyramided with *QFhs.nau-2DL* (Jiang et al., 2007; Agostinelli et al., 2012; Clark et al., 2016). Some other popular QTL widely used across wheat breeding programs include *Fhb3* for type II resistance translocated from chromosome *7Lr#1* of wild grass *L. racemosus* (Qi et al., 2008), *Fhb4* for type I resistance descended from the Chinese landrace Wangshiubai and mapped to *4BL* (Xue et al., 2010), *Fhb7* from *Th. ponticum* (Guo et al., 2015), and *Fhb7AC* on *7A* (Jayatilake et al., 2011) most of which are pyramided to *Fhb1*. A recent study on the *Fhb7* gene encoding a glutathione S-transferase reveals that broad resistance conferred to *Fusarium* species by *Fhb7* is due to xenobiotic detoxification of the trichothecene compounds as was observed in *Fhb1* (Wang et al., 2020). Further, the occurrence of the *Fhb7* gene in the wild wheat relative *Th. elongatum* genome might be through a “natural” fungus-to-plant horizontal gene transfer event (Wang et al., 2020; Wulff and Jones, 2020). Wheat breeding programs will benefit from successful introgression of *Fhb7* in diverse wheat backgrounds through distance hybridization conferring broad resistance to both FHB and crown rot.

### Phenological and Morphological Traits Associated With FHB Resistance

Buerstmayr et al. (2019) grouped FHB resistance mechanisms into two classes: one conferred by host plant resistance gene(s) and is widely considered in breeding programs (active resistance factors with five resistance types) and the other due to developmental and morphological traits (passive resistance factors) that mainly constitute plant stature, anther extrusion, and flowering date (**Figure 3**). Thus, QTL mapping should consider both customary FHB resistance as well as morphological traits associated with FHB resistance to enrich the genetic diversity for durable resistance. The potential of phenological and morphological traits to enhance FHB resistance has been currently deemed as a key to successful strategic breeding (Buerstmayr et al., 2019) although the importance of these traits on resistance expression was previously reported (Mesterhazy, 1995; Miedaner, 1997; Liu et al., 2013). A summary of QTL mapping studies conducted between 2009 and 2019 revealed that nearly 40% of the QTLs for plant height, 60% for anther extrusion, and 25% for flowering date overlapped with FHB resistance QTL (Buerstmayr et al., 2019). This could be due to linkage drag (tightly linked locus) or pleiotropy between morphological and disease traits.

Several studies have claimed that the semi-dwarf plants harboring *Rht-D1b* and *Rht-B1b* alleles are more prone to FHB epidemics (Buerstmayr et al., 2009; Mao et al., 2010; Steiner et al., 2017). Mesterhazy (1995) demonstrated that awnless, taller wheat plants are usually less infected compared to shorter plants with awns. A possible explanation could be that rain hitting soil/debris only splashes 40–60 cm high and thus taller plants would be escaped from infection. Wheat awns forming larger head surface could trap and funnel airborne conidia down to open florets and enhance infection. Flower morphology also affects FHB resistance where a narrow flower opening is more prone to FHB infection (Gilsinger et al., 2005). Buerstmayr and Buerstmayr (2016) observed increased FHB severity as an effect of the high proportion of retained anthers and reduced plant height associated with *Rht-D1b* and *Rht-B1b* where the former allele had a significantly higher impact compared to the latter. These ‘green revolution’ genes introduced to enhance grain yield were also found to have a significant association with FHB infection counting up to 41% of type I susceptibility and 13–23% of reduced anther extrusion (Miedaner and Voss, 2008; Perovic et al., 2008; He et al., 2016). Interestingly, Yi et al. (2018) underlined the unique genetic relationship of FHB QTL to morphological traits where spike compactness strongly affected FHB in point inoculation while plant height and spike compactness contributed more to FHB severity compared to days to flowering with spray inoculation.

### Modern Genomic Approaches for Breeding FHB Resistance

Modern breeding programs utilize novel technologies including transgenesis, gene cloning, and gene editing to complement conventional breeding for incorporation of durable resistance against FHB (Ma et al., 2020). The successful cloning of *Fhb1, 2DL*, and recently *Fhb7* and the development of diagnostic markers have contributed towards deployment of these QTL (Kage et al., 2017; Li et al., 2019; Su et al., 2019; Wang et al., 2020). Lagudah and Krattinger (2019) compared three recent contradictory papers on *Fhb1* gene cloning and suggested that both the Pore-forming toxin-like gene (Rawat et al., 2016) and Histidine-rich calcium-binding-protein gene (Li et al., 2019; Su et al., 2019) could possibly be contributing independently towards FHB resistance. The possibility of either dominant-negative model proposed by Lagudah and Krattinger (2019) or some unknown mechanism(s) of spatiotemporal gene function due to differential inoculation time (pre-anthesis, early anthesis, and anthesis) applied in all three studies is a debatable subject. Indeed, it could be a topic of beneficial scientific debate to gain further knowledge on the *Fhb1* gene complex and its use in breeding for FHB resistance (Hao et al., 2020).

Genes contributing to FHB resistance have been introduced from alien species using transgenic approaches for over 20 years (Ma et al., 2020). These include the pathogenesis-related (PR) gene, *ScNPR1* (*Secale cereale-NPR1*) from rye (Yu et al., 2017), overexpression of barley *HvUGT13248* and *Brachypodium distachyon UGT Bradi5gUGT03300* for improved detoxification of DON to D3G and NIV to NIV3G respectively (Li et al., 2015; Gatti et al., 2019), and introduction of barley class II chitinase gene for enhancing Type II and III resistance in wheat (Shin et al., 2008). Despite the daunting efforts, only limited success has been achieved to engineer transgenes in greenhouse environments, which also need further verification under field conditions to validate its functionality and genetic stability. Additionally, novel approaches such as expressed QTL (eQTL) combining transcriptomes data with conventional QTL mapping to identify genomic regions harboring candidate genes for FHB resistance have been initiated (Kazan and Gardiner, 2018). Similarly, host susceptibility genes could be explored to disable these deleterious genes through mutagenesis or editing through CRISPR/Cas9 (Wang et al., 2014). Host-induced gene silencing (HIGS) through the production of small interfering RNA molecules (RNAi) as demonstrated recently in wheat and model organism *B. distachyon* for FHB resistance could be further utilized (Cheng et al., 2015; He et al., 2019). However, regulatory hurdles have limited success for obtaining improved FHB resistant cultivars via a transgenic approach.

The success in sequencing whole genome of wheat has opened new avenues for screening multiple loci for FHB resistance at a much faster speed (IWGSC, 2014; Appels et al., 2018; Ma et al., 2020). Along with the readily available genomic data in public data repositories, there has been increased use of single nucleotide polymorphism (SNP) and microsatellite markers using modern next-generation sequencing (NGS) platforms such as genotyping-by-sequencing (GBS) and high-density Illumina 90K SNP assay in several QTL and association mapping projects (Deschamps et al., 2012; Liu et al., 2012; Islam et al., 2016; Petersen et al., 2016; Zhao et al., 2018). The sequence availability of both the *F. graminearum* and the wheat genome will allow greater understanding of the inherent mechanisms and genes underlying host–pathogen interactions contributing to effective management.

### Molecular Mechanisms of FHB Resistance

Recent advancement in molecular/genetic analysis along with the advent of genomic tools and modern technologies contributed substantially in partially understanding the mechanism of disease resistance. However, a comprehensive understanding of the complex network of cellular and molecular events and pathway involved in FHB resistance remains to be elucidated. Pritsch et al. (2000) initiated a study on the role of PR genes (*PR-1, PR-2, PR-3, PR-4*, and *PR-5*) on the resistance mechanism in response to *F. graminearum* infection and disease spread in Sumai 3. The study revealed that this basal defense response triggered as early as 6–12 hours after inoculation (hai) and peaked at 36–48 hai, much faster in resistant compared to susceptible cultivars. Later, Golkari et al. (2007) demonstrated that glume and rachis were the most responsive organs with the earliest transcriptome pattern observed at 24 hai. Since then, transcriptomic data has been widely explored to portray the potential mechanisms behind QTL-mediated defense responses against *Fusarium* species and elucidate the system-level understanding on the biology of the pathogen (Kazan and Gardiner, 2018; Ma et al., 2020). The successful combination of transcriptomics with metabolomics and proteomics approaches identified the defense-response functionality of the FHB resistance QTL *2D*, *Fhb1* and *Qfhs.ifa-5A* in wheat NILs (Warth et al., 2015; Kage et al., 2017).

Salicylic acid (SA) inducing systemic acquired resistance (SAR) has been perceived as a key element in early signaling events during FHB infection (Ma et al., 2020). Ding et al. (2011) observed the up-regulation of SA pathways with higher hormone accumulation in the resistant Chinese cultivar Wangshuibai inducing *PAL, EDS1, NPR1*, and *Glu2* genes within 3 hai. The early signaling pathways for SAR, mediated by coordinated and ordered expression of SA, jasmonic acid, ethylene, calcium ions, phosphatidic acid, and phenylpropanoid in addition to reactive oxygen species (ROS) production and scavenging, programmed cell death, cell wall fortification, and lignin biosynthesis have been widely discussed (Ding et al., 2011; Ma et al., 2020). The ability of the host plant to curb *F. graminearum* infection through the production of detoxification enzymes as observed in recent studies is another important approach to understand FHB resistance mechanisms (Lemmens et al., 2005; Gatti et al., 2019; Wang et al., 2020).

## Genetic Sources of FHB Resistance in the Southeast

Although initial efforts were mostly focused on mapping FHB resistance QTL in spring wheat due to the severe epidemics in the Northern Grain Plains in the 1990s (McMullen et al., 1997), increased FHB in the southeast has shifted breeding efforts on SRWW to include this disease. Seven QTL mapping studies consisting of eight mapping populations, recombinant inbred lines (RILs), doubled-haploids (DHs), or mapping panel with 98–256 lines were considered for FHB resistance (**Table 1**) (Liu et al., 2012; Liu et al., 2013; Islam et al., 2016; Petersen et al., 2016; Petersen et al., 2017; Tessmann and Van Sanford, 2018; Tessmann et al., 2019). The major sources of FHB resistant alleles in those studies were: VA00W-38, Massey, Becker, Ernie, MO 94-317, NC-Neuse, and Truman. All the mapping studies considered type I–IV resistance in both greenhouse and field-based studies. Except for one study (Islam et al., 2016), all other mapping studies assessed morphological and phenological traits to explore their association with FHB traits. A total of 76 QTLs were detected across seven mapping studies of which 28 were considered as major effect QTLs (11, 10, and seven on chromosomes A, B, and D respectively). Since a single QTL was contributing to more than one FHB trait implying that the FHB QTL has a pleiotropic effect, the studies found a total of 10 major QTLs associated with type I, 10 with type II, 6 with FHB Index, 13 with type III, and 17 with type IV resistance. The identification of some major effect QTL for type III and IV resistance observed in recent years in the SRWW region encouraged the wheat breeders in the southeast US to screen wheat varieties with major available QTLs associated with low DON and FDK. Further, the higher number of type III and type IV resistance QTL reported in these studies is in contrast to the highest number of type II QTL reported in a review by Venske et al. (2019) showcasing the interest shift on FHB traits in this region. Furthermore, it was evident that the lower number of QTLs was detected on a small mapping population, while the number of QTLs increased for the large-sized population as mentioned by Buerstmayr et al. (2009). A higher number of FHB traits were associated with QTL when studies were carried out both in the greenhouse and field as compared to field studies alone. This could be possible as low heritable FHB QTLs are only fully expressed when populations are tested across multiple environments for multiple years (Mesterházy et al., 1999). Interestingly, the association of FHB QTL with plant height and heading date observed in most of the studies corroborates the necessity to consider these phenotypes in QTL mapping projects (Miedaner and Voss, 2008; He et al., 2016; Buerstmayr et al., 2019). For instance, out of the four common QTLs associated with FHB variables in both Becker/Massey and Ernie/MO 94–317 mapping populations, three of them overlapped with genes governing plant height (*Rht*-*B1* and *Rht*-*D1*) and photoperiod sensitivity (*Ppd*-*D1*) (**Table 1**) (Liu et al., 2013). The pleiotropic effects of these morphological genes suggest that deploying and pyramiding FHB resistance QTL with these important morphological traits could be an effective strategy to improve FHB resistance in SRWW cultivars. Further, the low phenotypic variation (−2.1 to 26.7%) explained by QTL identified in the SRWW panels and mapping population is not unusual for polygenic nature of FHB resistance where multiple small effects QTL contribute to resistance (Petersen et al., 2017; Tessmann et al., 2019).

**Table 1:** Summary of QTL mapping studies for FHB resistance in SRWW in the southeast US.

Mapping Population/panel	Population size^a^	Number of experiments^b^	Inoculation techniques^c^	FHB traits assessed^d^	Associated morphological/phenological traits assessed^e^	Source of resistance allele	Chromosome allocation of all detected QTL	Major QTL/chromosome with associated traits	Linked markers for major QTL	*R^2f^*	References
VA00W-38/Pioneer brand ‘26R46’	182 RILs	Field (4)	SI	INC, SEV, IND, FDK, DON	FT or HD	VA00W-38	*1BL, 2AS, 2ASc, 2AL, 2DL, 5B, 6A, 7A*	*2DL* (INC), *5B* (INC, DON), *6A* (FDK)	*Xgwm349* for *2DL*; *Xgwm271* for *5B*	5.7–21.3	Liu et al., 2012
Becker/Massey	152 RILs	GH (4); Field (7)	SFI (GH); SI (Field)	INC, SEV, IND, FDK, DON	FT (photoperiod sensitivity), PHT	Massey & Becker	*1AS, 1DS, 2DS, 2BL, 3BL, 4BS, 4DS, 4DL, 6BL, 7A*	*2DS* (INC, IND), *4BS* (INC, IND, FDK), *4DS* (INC)	*Xgwm261, Xgwm484* for *2DS*; *Xgwm513, Xgwm149* for *4BS*; *Xbarc334, Xgwm192* for *4DS*	–	Liu et al., 2013
Ernie/MO 94-317	231 RILs	GH (2); Field (5)	SFI (GH); SI (Field)	INC, SEV, IND, FDK, DON	FT (photoperiod sensitivity), PHT	Ernie & MO 94-317	*2AL, 2BL, 2DS, 3BL, 4BS, 4DS, 5AL, 6AL*	*2DS* (SEV, IND), *4BS* (INC, SEV, IND, FDK), *4DS* (INC, SEV, IND, FDK, DON), *5AL* (INC, SEV, IND)	*Xgwm261, Xgwm484* for *2DS*; *Xgwm513, Xgwm149* for *4BS*; *Xbarc334, Xgwm192* for *4DS*; *Xgwm291* for *5AL*	–	Liu et al., 2013
NC-Neuse/AGS2000	170 RILs	Field (7)	CS	INC, SEV, FDK, DON	PHT, HD	NC-Neuse	*1A, 1B, 1D, 2A, 4A, 5B, 6A*	*1A* (INC, SEV, FDK, DON), *1B* (FDK, DON), and *6A* (INC, SEV)	KASP markers *Qfhb.nc‐1A* for *1A; Qfhb.nc‐1B* for *1B; Qfhb.nc‐6A* for *6A*	5.7–19.5	Petersen et al., 2016
Truman/MO 94-317	167 RILs	GH (2); Field (2)	SFI (GH); SI or CS (Field)	INC, SEV, IND, FDK, DON	–	Truman	*1BSc, 1BL, 1DLc, 2ASc, 2BL, 2DS, 3AL, 3BSc, 3BL, 3DS*	*2ASc, 2DS, 3DS* (SEV, FDK, DON for all locus)	*wPt8826* for *2ASc; wPt666223* for *2DS; wPt5390 for 3DS*	6.7–25.3	Islam et al., 2016
Truman/NC-Neuse	98 DHs	Field (7)	CS, SI	INC, SEV, FDK, DON	PHT, HD	NC-Neuse, Truman	*1A, 1B, 2A, 2B, 3B, 4A.2a, 4A.2b, 4D, 5B, 5D, 6A2, 6A.3*	*2B* (SEV, FDK, DON)	KASP marker *Qfhb.nc-2B.1*	6.2–26.7	Petersen et al., 2017
											
Mapping panel	236 SRWW cultivars/breeding lines	Field (2)	SI, CS	FHB rating (0-9 scale), FDK, DON	PHT, HD	–	*1A, 1B, 1D, 2A, 3B, 3D, 4A, 5A, 5B, 5D, 6A, 6B, 7A, 7B*	*1A, 1B, 2A, 3B, 5A* (FDK), *4A, 6A, 6B* (DON)	–	0.08–0.19	Tessmann and Van Sanford, 2018
											
Mapping panel	256 SRWW cultivars/breeding lines	Field (2)	CS	FHB rating (0-9 scale), INC, SEV, IND, FDK, DON	PHT, HD	–	*3A, 4A, 5B, 6A, 6B, 7A, 7B*	*4A* (DON), *5B* (FDK, DON), *6B* (FDK)	–	−2.1–4.0	Tessmann et al., 2019

^a^RILs, recombinant inbred lines; DHs, double-haploids; SRWW, soft red winter wheat.

^b^GH, greenhouse.

^c^SI, spray inoculation (conidial); SFI, single floret inoculation; CS, corn spawn.

^d^INC, disease incidence; SEV, disease severity; IND, FHB Index; FDK, Fusarium-damaged kernel; DON, deoxynivalenol.

^e^FT, flowering time; HD, heading date; PHT, plant height.

^f^The highest amount of phenotypic variation (%) explained by the QTL.

QTL validation is another important step towards the development of resistant cultivars. Four studies on QTL validation were conducted in the southeast US using eleven mapping populations developed as back-crossed lines, NILs or RILs (**Table 2**) (Chen et al., 2006; Kang et al., 2011; Balut et al., 2013; Clark et al., 2016). These studies had either W14 or Sumai 3 as a donor parent and validated *Fhb1 (3BS), QFhs.nau-2DL*, and *5AS (Fhb5*) QTLs in diverse backgrounds. Most of the lines in these studies developed by marker-assisted backcrossing and selection highlight the wider application of MAS in the southeast for screening and developing wheat cultivars. As an instance, three FHB QTL *3BS* (*Fhb1*), *2D*, and *5A* from non-adapted Chinese cultivar Ning7840 was pyramided into SRWW cultivar McCormick by marker-assisted backcrossing (Kang et al., 2011). Further, the exotic and native QTLs were deployed from Ning7840 and McCormick, respectively, to develop SRWW germplasm KY06C-11-3-10 using the same approach (Clark et al., 2014). This germplasm has been further used as a donor parent for developing breeding populations. Chen et al. (2006) confirmed the presence of major FHB resistance QTLs on chromosomes *3BS* and *5AS* in Chinese line W14 contributed to reduced initial infection, disease spread, kernel infection, and DON accumulation. The use of double-haploid populations in this study has shortened the breeding cycle thereby expediting the validation and further QTL deployment processes. Overall, greater emphasis is ongoing to introgress FHB resistance QTL both from exotic and native sources in current SRWW cultivars through efficient breeding approaches such as genomic selection and hastily using DH method.

**Table 2:** Summary of main QTL validation studies for FHB resistance in SRWW in the southeast US.

Mapping Population	Population size^a^	Number of experiments^b^	Inoculation techniques^c^	Targeted FHB traits^d^	Associated morphological/phenological traits^e^	Resistant donor	Validated QTL/chromosome	Markers used for screening	References
W14/Pion2684	96 DHs	GH (2); Field (1)	SFI (GH); SI (Field)	INC, SEV, FDK, DON (for both QTL)	–	W14	*3BS (Fhb1), 5AS (Fhb5)*	*Xbarc133, Xgwm493* for *3BS; Xbarc117, Xbarc56* for *5AS*	Chen et al., 2006
McCormick/Ning7840	8 NILs using marker-assisted backcrossing	GH (1); Field (3)	SFI (GH); CS (Field)	INC, SEV, FDK, DON (*2DL* and *3BS*)	–	Ning7840 (Sumai 3)	*3BS (Fhb1), 2D, 5A*	*Xgwm533*, *Xcfd79, XUMN10* for *Fhb1*; *Xgwm304, Xbarc186* for *5A*; *Xgwm539, Xgwm608* for *2D*	Kang et al., 2011
5 different populations	155 RILs	Field (2)	CS	INC, SEV, IND, FDK, DON	Agronomic, milling, and baking quality	W14	*Fhb1, QFhs.nau-2DL*	*Xgwm533* for *Fhb1; Xcfd233* for *QFhs.nau-2DL*	Balut et al., 2013
(4 populations) KY97C-0321-05-2/VA01W-476; KY97C-0519-04-05/VA01W-476; KY97C- 0540-01-03/VA01W-476; KY97C-0508-01-01A/VA01W-476	379 lines from all 4 crosses (BC_1_F_1:3_ and BC_1_F_1:4_)	Field (2)	CS	INC, SEV, IND, FDK, DON	PHT, HD, milling, and baking quality	W14	*Fhb1, QFhs-nau-2DL*	*UMN10, gwm533* for *Fhb1; cfd233, gwm539* for *QFhs.nau-2DL*	Clark et al., 2016

^a^RILs, recombinant inbred lines; DHs, double-haploids; NILs, near isogenic lines; BC, backcrossing.

^b^GH, greenhouse.

^c^SI, spray inoculation (conidial); SFI, single floret inoculation; CS, corn spawn.

^d^INC, disease incidence; SEV, disease severity; IND, FHB Index; FDK, Fusarium-damaged kernel; DON, deoxynivalenol.

^e^HD, heading date; PHT, plant height.

In the southeast US, each participating program routinely screens for FHB resistance as an important criterion before releasing new wheat cultivar in the region. The elite lines are screened for FHB resistance in the SUWWSN (https://scabusa.org/) and Cooperative SRWW Nurseries (consisting of 6-State Advanced and Preliminary (6ST-ADV & 6ST-PRE), Gulf Atlantic Wheat (GAWN) and Sun Wheat nurseries) for multiple years. These lines are also genotyped in the USDA Eastern Regional Small Grains Genotyping Laboratory at Raleigh, NC to identify robust markers associated with FHB resistance traits. The most common resistance loci found across SRWW and breeding lines in SUWWSN recently include *Fhb1, Fhb_3B_Massey, Fhb_5A_Ernie, Fhb_5A_Ning, Fhb_2DL_Wuhan1/W14, Fhb_1B_Jamestown, Fhb_1A_Neuse, Fhb_4A_Neuse, Fhb_6A_Neuse, Fhb_2B_Bess*, and *Fhb_3B_Bess* (Murphy et al., 2019*)*. Breeding materials are also tested in the regional Uniform Eastern & Southern SRWW Nurseries and screened for all these resistance loci except *Fhb_2DL_Wuhan1/W14*. A detailed list of the SSR and SNP markers used in the genotypic analysis can be found at: https://www.ars.usda.gov/southeast-area/raleigh-nc/plant-science-research/docs/small-grains-genotyping-laboratory/regional-nursery-marker-reports/. Notably, the registration of FHB resistant SRWW germplasm NC-Neuse (Murphy et al., 2004), Jamestown (Griffey et al., 2010a), VA04W-433, VA04W-474 (Chen et al., 2012), KY06C-11-3-10 (Clark et al., 2014), and AGS 3015 (Mergoum et al., 2019) is among the FHB breeding achievements obtained so far in the region (see **Table 3** for representative list of SRWW cultivars).

**Table 3:** List of representative major FHB and rusts resistant SRWW cultivars/germplasm released in the southeast US.

SRWW cultivars/germplasm	Pedigree	Resistance against FHB^a^	Resistance against LR^b^		Resistance against SR^c^	QTL/chromosome^d^	Year of registration	Institution(s) involved	References
Jaypee	‘Doublecrop’/AR 39-3	–	✓		✓	*Lr10, Lr18*	1995	Arkansas Agricultural Experiment Station	Bacon et al., 1998
Roane	VA 71-54-147/’Coker 68-15’//IN65309C1-18-2-3-2	✓	✓		–	*Lr10, Lr11*	1999	Virginia Agricultural Experiment Station	Griffey et al., 2001
NC-Neuse	Coker 86-29//’Stella’/CHD 756-80/3/’Coker 9907’	✓	✓		–	*Lr9, Lr10, Lr11*	2003	North Carolina Agricultural Research Service	Murphy et al., 2004
McCormick	VA92-51-39/AL870365	✓	✓		✓	*Lr24*	2002	Virginia Agricultural Experiment Station	Griffey et al., 2005a
Tribute	VA92-51-39/AL870365	✓	✓		✓	*Lr9, Lr24*	2002	Virginia Agricultural Experiment Station	Griffey et al., 2005b
Allegiance (KY90C-054-6)	Pioneer Brand ‘2548’/’SS 555’	✓	✓		✓	*Lr10*	2002	Kentucky Agricultural Experiment Station	Van Sanford et al., 2006
Chesapeake	VA91-54-222 (‘Roane’”S”)/’FFR555W’//VA93-52-55	–	✓		–	*Lr26*	2005	Maryland Agricultural Experiment Station and Virginia Agricultural Experiment Station	Costa et al., 2007
5205 (VA01W-205)	Pioneer Brand ‘2684’/VA93-54-185//’Pocahontas’	✓	✓		✓	*-*	2008	Virginia Agricultural Experiment Station	Griffey et al., 2009a
USG 3555 (VA02W-555)	VA94-52-60/Pioneer Brand ‘2643’//’USG 3209’	✓	✓		✓	*Lr11, Lr26*	2007	Virginia Agricultural Experiment Station	Griffey et al., 2009b
Jamestown	‘Roane’/Pioneer Brand ‘2691’	✓	✓		✓	*1B; Lr10, Lr18*	2007	Virginia Agricultural Experiment Station	Griffey et al., 2010a
Shirley (VA03W-409)	VA94-52-25/’Coker 9835’ (PI 548846 PVPO)//VA96–54-234	✓	✓		–	*-*	2008	Virginia AgriculturalExperiment Station	Griffey et al., 2010b
SW049029104	‘38158’ (PI 619052)/Pioneer brand 2552 (PI 566924)//’Roane’ (PI 612958)	✓	✓		–	*Lr11*	2009	Virginia Agricultural Experiment Station	Griffey et al., 2011
VA04W-433	Ning7840 (PI 531188)/Pioneer brand ‘2684’ (PI566923)//VA96-54-244	✓	✓		–	*3BS*	2009	Virginia Agricultural Experiment Station	Chen et al., 2012
VA04W-474	‘Roane’ (PI 612958)//W14(PI 641164)/’Coker 9134’ (PI 573034PVPO)	✓	✓		–	*5AS*	2009	Virginia Agricultural Experiment Station	Chen et al., 2012
KY06C-11-3-10	Ning7840’/’McCormick	✓	✓		✓	*Fhb1, 5A, 2DL; Lr34, Lr24; Yr18*	2013	Kentucky Agricultural Experiment Station, Maryland Agricultural Experiment Station, Virginia Polytechnic Institute and State University, North Carolina State University, and the USDA–ARS	Clark et al., 2014
Pembroke 2014	‘25R18’/KY92C-0010-17//KY96C-0767-1	✓	–		✓	*Fhb1*	2014	Kentucky Agricultural Experiment Station	Van Sanford et al., 2016
Hilliard	‘25R47’/’Jamestown	✓	✓		✓	*-*	2015	Virginia Agricultural Experiment Station	Baik et al., 2017
GA 03564-12E6	SS 8641/4/’AGS2000’*3/931433//’2684’/3*AGS 2000	–	✓		✓	*Lr37; Yr17*	2015	UGA small grain breeding program and SUNGRAINS	Johnson et al., 2017
Pembroke 2016	‘Pioneer 25W33’/’Pioneer 25W60’//Pioneer 25W33/KY90C-042-37-1	✓	✓		✓	*-*	2016	Kentucky Agricultural Experiment Station	Van Sanford et al., 2018
Savoy	GA 931233/’USG3592’//GA 941208-2E35	–	✓		✓	*-*	2015	UGA small grain breeding program and SUNGRAINS	Johnson et al., 2018
AR11LE24 (AGS 2055)	GA961591-3E42/GA96229-3A41	–	✓		✓	*-*	2015	University of Arkansas’s Division of Agriculture, UGA, and SunGrains	Mason et al., 2018
GA09129-16E55 (AGS 3015)	991109-6E8/IL00-8530//991109-6E8	✓	✓		✓	*1A, 1B, 4A, 6A; Lr37; Yr17*	2019	University of Georgia small grain breeding program	Mergoum et al., 2019

^a,b,c^(✓) indicates SRWW resistance to FHB, LR, and SR, respectively.

^d^QTL resistance for FHB, LR, and SR is listed in order and each category is separated by a semi-colon (;).

## Genetics of LR and SR Resistance and Breeding Strategies

### Types of Resistance

Resistance to rust diseases in wheat is broadly classified into two categories; seedling and adult plant resistance (APR) (Kolmer, 1996; Chen, 2007; Kolmer et al., 2007). Seedling resistance, also known as all-stage resistance, is detected in the seedling stage and remains effective at all stages of plant growth. Seedling resistance is race-specific, controlled by a single gene and mostly effective for a short duration due to the continuous evolution of new races. In contrast, APR is detected at the adult stage, controlled by multiple genes with a small effect, and is typically more durable (Line and Chen, 1995). APR genes are further classified into race-specific and race non-specific resistance genes (Ellis et al., 2014). The majority of the winter wheat cultivars possess high-temperature adult plant (HTAP) resistance typical to SR which is expressed only at the high post-inoculation temperatures (Qayoum and Line, 1985). Wheat cultivars that possess only HTAP resistance are susceptible at the seedling stage when the temperature is low, but as the temperature increases, the level of their resistance also increases (Line and Chen, 1995; Chen, 2007; Ellis et al., 2014). Research evidence proved that race non-specific HTAP resistance mostly triggers at the jointing and later growth stages with flag leaves exhibiting more resistance than the lower leaves within the same plant (Qayoum and Line, 1985). Moreover, SR development was much slower on highly resistant cultivars establishing direct correlation of rust development with HTAP resistance. Apart from seedling and APR, wheat cultivars were also found to have slow rusting or partial resistance to LR and SR (Line and Chen, 1995; Singh et al., 2005; Figlan et al., 2020). Similar to APR, slow rusting resistance is controlled by multiple genes with small effects that are race non-specific and more durable (Kolmer, 1996).

### Sources of Resistance

A wild relative of wheat, Sharon goatgrass (*Aegilops sharonensis*), native to the fertile crescent region, has long been considered a possible source of unique resistance genes to several wheat diseases with the highest resistance frequency (60–77%) observed for LR and intermediate resistance (45%) for SR (Olivera et al., 2007; Olivera et al., 2008; Millet et al., 2014). Today, several other wild relatives of wheat contributing a diverse genetic pool against rust resistance are widely exploited. Some of the successful transformation from donor relatives documented include major resistance genes *Lr21, Lr22a*, and *Lr39* from Tausch’s goatgrass (*Ae. tauschii*) (Raupp et al., 2001; Kolmer, 2013), *Lr24* from wheatgrass (*Thinopyrum ponticum*) (Li et al., 2003), *Lr57* from *Ae. geniculate* (Kuraparthy et al., 2007), *Lr37/Yr17* from *Ae. ventricosa* (Kolmer, 2013), *Lr9* and *Lr26/Yr9* from *Ae. umbellulata* and common rye (*Secale cereal*) respectively (Browder, 1980), *Yr15* from *T. dicoccoides* (Gerechter-Amitai et al., 1989), and *Yr8* from *Ae. comosa* (Friebe et al., 1996). Cryptic alien introgressions may provide a unique source of resistance but are also likely to bring additional undesirable or even deleterious genes that reduce cultivar fitness, quality, and even grain yield due to linkage drag (Kuraparthy et al., 2007).

### QTL Mapping for LR and SR Resistance and Validation

Similar to FHB, biparental QTL mapping using two divergent cultivars was instrumental in identifying rust resistance loci. Currently, 79 *Lr*-genes (*Lr1*-*Lr79*) and 82 *Yr*-genes have been formally cataloged from common wheat, durum wheat, and other related species (McIntosh et al., 2017; Qureshi et al., 2018; USDA Cereal disease lab, https://www.ars.usda.gov/midwest-area/stpaul/cereal-disease-lab/docs/resistance-genes/resistance-genes/). Of the 79 *Lr*-genes and 82 *Yr*-genes, 64 and 15 *Lr*-genes, and 59 and 19 *Yr*-genes confer seedling and APR, respectively (McIntosh et al., 2017; Da Silva et al., 2018; Sapkota et al., 2019a). Among the 15 *Lr*-genes and 19 *Yr*-genes for APR, seven (*Lr12*, *Lr13*, *Lr22a/b*, *Lr35*, *Lr37*, *Lr48*, and *Lr49*) and eight (*Lr34*, *Lr46*, *Lr67*, *Lr68*, *Lr74*, *Lr75*, *Lr77*, and *Lr78*) *Lr*-genes and five (*Yr11, Yr12, Yr13, Yr14*, and *Yr34*) and fourteen (*Yr16, Yr18, Yr29, Yr30, Yr36, Yrns-B, YrA1, YrA2, YrA3, YrA4, YrA5, YrA6, YrA7*, and *YrA8*) *Yr*-genes are reported to confer race-specific and race non-specific resistance, respectively (McIntosh et al., 2017; Da Silva et al., 2018; Waqar et al., 2018).

QTL mapping of slow rusting genes expressing a prolonged latent period has gained scientific interest to assure durable resistance to wheat LR. For example, the winter wheat line CI13227, well known for slow rusting resistance, has been widely used in mapping studies. Several research groups have demonstrated that CI13227 has both seedling resistance gene *Lr3ka* as well as APR genes on *1BL* likely to be *Lr46* along with *2B, 2DS, 7AL*, and *7BL* (Xu et al., 2005a; Xu et al., 2005b; Kolmer et al., 2012; Kolmer et al., 2015). The seedling resistance genes found in CI13227 might have contributed to the development of small uredinia, lower field infection, and a longer latent period possibly attributing additive effects with the APR genes (Kolmer et al., 2012). Later, Kolmer et al. (2015) found that *Lr46* gene additionally confers APR to SR, stem rust, and powdery mildew. Another major effect QTL *Lr74* mapped to *3BS* region was detected in two SRWW cultivars Clark and Caldwell and is considered an important genomic region that contains stem rust resistance gene *Sr2* and FHB QTL *Fhb1* (Li et al., 2017; Kolmer et al., 2018). Further studies to determine if *Lr74* is linked in coupling phase with *Sr2* as in the case for *Sr2* and *Fhb1* would assist in combining these genes in breeding programs (Li et al., 2017). In addition, the presence of seedling resistance genes *Lr9, Lr10*, and *Lr14a* in Coker 9663 and *Lr14b* and *Lr26* in Pioneer 26R61 together with APR gene *Lr13* in the latter demonstrates the availability of copious genetic resources across SRWW cultivars (Kolmer, 2010).

With increasing numbers of QTL mapping studies, the identification of closely linked markers and their validation for MAS is indispensable. Validation of molecular markers for some of the LR resistance genes such as *Rph5* and *Rph7* in barley is noteworthy in terms of its applicability in gene postulation, MAS, and eventual gene pyramiding with other resistance genes (Mammadov et al., 2007). Similarly, several breeding approaches have been carried out to develop diagnostic markers that are tightly linked to LR resistance locus in wheat. Dedryver et al. (1996) developed a sequence characterized amplified region (SCAR) marker converted from a random amplified polymorphic DNA marker, OP-H5, linked to *Lr24* locus indicating the possibility of molecular screening of LR resistance through MAS. Gene pyramiding for gaining durable LR resistance is usually effective through MAS once the diagnostic markers are developed and validated in diverse backgrounds. Kloppers and Pretorius (1997) revealed that wheat lines with combinations of *Lr34* (race non-specific) and *Lr13* (race-specific) as well as *Lr34* and *Lr37* genes showed improved resistance exhibiting active complementary effect even in the presence of LR races possessing virulence for *Lr13* gene. CIMMYT has been working for an extended period of time to pyramid additive slow rusting LR and SR genes (usually four or five) having small to intermediate effects in locally adapted wheat cultivars through backcrossing breeding approach (Singh et al., 2005). In this light, several kinds of PCR-based molecular markers, including SCAR, AFLP, and cleaved amplified polymorphic sequences (CAPS), are available to screen for LR resistance genes *Lr1, Lr9, Lr10, Lr19, Lr21, Lr24, Lr25, Lr28, Lr29, Lr34, Lr35, Lr37, Lr39, Lr47*, and *Lr51* and SR resistance genes *Yr10, Yr15, Yr17, Yr18*, and *Yr32* further assisting in gene pyramiding (Robert et al., 1999; Spielmeyer et al., 2005; Murphy et al., 2009; Todorovska et al., 2009).

### Modern Genomic Approaches for Breeding LR and SR Resistance

Limited success has been achieved by using phenotyping screening, physiologic specification of virulence races and conventional QTL mapping studies to breed wheat cultivars with durable LR and SR resistances. Similar to the approach for FHB resistance, a system-based approach understanding the host plant and their obligate parasites can help to develop resilient breeding strategies. Development of transgenic wheat and exploiting HIGS still need to be largely explored (Figlan et al., 2020). Yin et al. (2011) summarized the ability of RNAi technology to suppress the expression pattern of SR fungus gene through the use of *barley stripe mosaic virus* system. The presence of RNAi triggering the downregulation of protein kinase A gene *PsCPK1*, an important pathogenicity factor, was found to enhance resistance to wheat SR (Qi et al., 2018). The expression of hairpin RNAi constructs homologous to *Pt* MAP-kinase (*PtMAPK1*) encoding genes resulted in the silencing of corresponding genes in LR fungi opening an alternative avenue for developing rust-resistant wheat cultivars (Panwar et al., 2018). Further, gene-deployment strategies to gain durable rust resistance require better understanding of potential pathogen variability. The emergence of new rust races can be constantly scouted by using sophisticated surveillance technology called “field pathogenomics” which uses RNAseq or genomic DNA-based approaches to generate high-resolution NGS data for describing pathogen dynamics (Derevnina and Michelmore, 2015; Figlan et al., 2020). These modern tools show considerable promise to provide insights into the biology, population structure, and pathogenesis of wheat SR and could help formulate breeding decisions while deploying rust resistance genes in SRWW cultivars (Hubbard et al., 2015).

Map-based cloning of rust resistance genes is still an arduous endeavor due to the polyploidy complex, large wheat genome size, and high content of repetitive DNA (Cloutier et al., 2007). Nonetheless, some LR resistance genes have been successfully cloned such as *Lr1* (Cloutier et al., 2007), *Lr10* (Feuillet et al., 2003), *Lr21* (Huang et al., 2003), *Lr22a* (Thind et al., 2017), *Lr34* (Krattinger et al., 2009), and *Lr67* (Moore et al., 2015). Similarly, SR resistance genes that have been cloned include *Yr5/YrSP* and *Yr7* (Marchal et al., 2018), *Yr10* (Liu et al., 2014), *Yr15* (Klymiuk et al., 2018), *Yr18* (Krattinger et al., 2009), *Yr36* (Fu et al., 2009), and *Yr46* (Moore et al., 2015). *Yr10* is considered the first seedling resistance gene cloned while other genes such as *Yr18/Lr34/Sr57/Pm38*, and *Yr46/Lr67/Sr55/Pm39* possess multi-pathogen (partial) slow rusting APR functionality against the three wheat rusts and powdery mildew (Spielmeyer et al., 2005; Krattinger et al., 2009; Moore et al., 2015; Klymiuk et al., 2018; Figlan et al., 2020). Despite the paucity of success in gene cloning, pathways to engineer genes of interest *via* high throughput modern genome editing technology have already been unlocked (Figlan et al., 2020) tracking success in powdery mildew resistance (Wang et al., 2014). Other modern genomic approaches to rust resistance gene isolation such as mutant chromosome sequencing (MutChromSeq), resistance gene enrichment sequencing (RenSeq), and platform combining association genetics (GWAS) with RenSeq (AgRenSeq) have been discussed recently (Dinh et al., 2020). Above all, the molecular and genomic basis of resistance breeding relies on our understanding of host resistance as well as pathogen virulence genes and their interactive mechanisms involved in rust resistance.

### Molecular Mechanisms of LR and SR Resistance

Plants defense mechanisms against pathogens are broadly classified into two categories *i.e.*, basal defense and R-gene mediated defense mechanism (Dinh et al., 2020). Basel resistance is effective against a wider range of plant pathogens and includes both host and non-host resistance; whereas, R-gene mediated resistance is effective against specific pathogens (Gururani et al., 2012; Dinh et al., 2020). Overall, rust disease resistance mechanisms in wheat take place in three main steps: recognition of the pathogens, signal transduction, and defense responses (Dinh et al., 2020). When the rust pathogens land on the wheat plant, pattern recognition receptors (PRRs) in the host plant detect the pathogens which is followed by the transduction of signals to the immune system of the plants. Once the pathogen is recognized and the signal is received, different mechanisms are induced in plant cells including programmed cell death (PCD) or sacrificing the infected cells and blocking the nutrient sources to the pathogens, as a disease resistance mechanism (Dinh et al., 2020).

Cereal hosts restrict the invasion and growth of biotrophic rust pathogens using two broad strategies, penetration resistance and PCD-mediated resistance (Gebrie, 2016). Penetration resistance occurs when non-adapted pathogens fail to recognize the plant’s physical and chemical signals necessary for subsequent infection. PCD-mediated resistance occurs inside the penetrated epidermal cell where the plant terminates nutrient supply to the fungal pathogens restricting further development by induction of invaded program cell death. Wheat resistance responses to rust pathogens have been characterized into surface-localized pathogen-associated molecular patterns (PAMPs)-triggered immunity (PTI), intracellular effector-triggered immunity (ETI), and broad-spectrum quantitative (partial) disease resistance (Dangl et al., 2013; Gebrie, 2016; Klymiuk et al., 2018). Dinh et al. (2020) discussed the significance of PAMPs conserved across plant pathogens that are recognized by PRRs in host plants. One of the well-known PAMPs present in cereal rust fungi is chitin (Dinh et al., 2020). Recognition of PAMPs triggers multiple defense responses in the host plant including generation of ROS, biosynthesis of defense hormones and phytoalexin, and cell wall strengthening which eventually generate PTI response (Gebrie, 2016; Dinh et al., 2020). The second layer of plant defense response, ETI, uses resistance (R) proteins that are activated upon the recognition of pathogen effectors (Dangl et al., 2013). The association of ETI with a hypersensitive response leading to PCD prevents rust pathogens from acquiring nutrients from cereal hosts (Gebrie, 2016; Dinh et al., 2020). Most of the cloned LR resistance genes, such as *Lr1, Lr10, Lr21*, and *Lr22a*, encode nucleotide-binding site leucine-rich repeat (NBS-LRR) proteins (Feuillet et al., 2003; Huang et al., 2003; Cloutier et al., 2007; Dinh et al., 2020). However, only a few of the SR resistance genes, such as *Yr10*, *Yr5/YrSP*, and *Yr7* encode this protein (Liu et al., 2014; Marchal et al., 2018). The activation of these NBS-LRR genes normally induces PCD or produces ROS in plants (Qi and Innes, 2013; Dinh et al., 2020). Therefore, the presence of these NBS-LRR genes in wheat is considered to be important to confer resistance against LR and SR with hypersensitive reaction *via* PCD.

## Genetic Sources of LR and SR Resistance in the Southeast

Significant progress has been made on developing SRWW cultivars with varying degrees of rust resistance (Kolmer, 2019). Almost all breeding programs that work on FHB resistance as mentioned before are actively involved in breeding LR and SR resistance SRWW cultivars in the southeast US. The UGA small grain breeding program in collaboration with the USDA-ARS Cereal Rust Laboratory registered six LR resistant SRWW germplasm in 1991 adapted to the southeast US: Ceruga-1, Ceruga-2, Ceruga-3, Ceruga-4, Ceruga-5, and Ceruga-6. This was the result of a cooperative research program initiated in 1982 (Long et al., 1992). Subsequently, most released SRWW cultivars in the southeast US have good resistance to LR and SR. At UGA, LR resistant cultivars recently released include AGS 2027, PIO 26R94, and SS 8629 (2013), AGS 2024, Savoy, and SH 0555 (2014), AGS 2033 (2015), Fury (Progeny 16-1) and AGS 2055 (2016), GW 2032, AGS 3030, and AGS 3040 (2017), USG 3640 (2018), and AGS 3015, Dyna-Gro Rutledge and Blanton (2019) most of which have *Lr37* resistance gene in their background (M. Mergoum, *personal communication and unpublished data*). The most common LR and SR resistance genes reported in SRWW cultivars for the southeastern states include the combination of *Lr1, Lr2a, Lr9, Lr10, Lr11, Lr12, Lr14a, Lr18, Lr26*, and* Lr37* and *Yr17* and *YrR6*, respectively (Kolmer, 2002; Hao et al., 2011; Kolmer, 2013; Kolmer, 2019).

Notably, gene postulation studies in SRWW cultivars and breeding lines also revealed the presence of seedling resistance genes *Lr1, Lr2a, Lr9, Lr10, Lr11, Lr18*, and *Lr26* and APR genes *Lr12* and *Lr34* when tested in the field of North Carolina (Kolmer, 2003). However, cultivars with *Lr2a, Lr9*, and *Lr26* genes in combination with APR genes *Lr12* and *Lr34* only exhibited higher resistance to LR. Additionally, gene postulation studies on 116 contemporary SRWW suggested the presence of seedling resistance genes *Lr1, Lr2a, Lr2c, Lr3, Lr3ka, Lr9, Lr10, Lr11, Lr14a, Lr18, Lr20, Lr23, Lr24*, and *Lr26* (Wamishe and Milus, 2004a) as well as the contribution of either *Lr12, Lr13*, or *Lr34* for APR to LR (Wamishe and Milus, 2004b). Recent reports on a genotypic analysis of SRWW breeding lines also revealed the presence of LR and SR resistance genes *Lr34/Yr18 (*on *7DS), Yr17/Lr37/Sr38 (*2NS:2A translocation*), Lr9 (*on *6BL), Lr18 (*from *T. timopheevii* on *5BL), Sr24/Lr24 (*introgressed from *Agropyron elongatum* on *3DS* or *1RS)*, and *Yr_4BL* (2019 Uniform Eastern & Southern SRWW Nursery Marker Report). The detailed list on different diagnostic markers used in the analysis can be found at: https://www.ars.usda.gov/southeast-area/raleigh-nc/plant-science-research/docs/small-grains-genotyping-laboratory/regional-nursery-marker-reports/. Some of the most common SRWW cultivars/germplasm lines registered and/or released for the southeast with LR and SR resistance include CK9553 and AGS 2056 (*Lr11*), AGS 2000 and Arcadia (*Lr26*), Jamestown and Hilliard (*Lr10*, *Lr18*), KY06C-11-3-10 (*Lr34*/*Lr24*/*Yr18*), and GA 03564-12E6 and AGS 3015 (*Lr37*/*Yr17*) (Clark et al., 2014; Baik et al., 2017; Johnson et al., 2017; Kolmer, 2019; Mergoum et al., 2019) (see **Table 3** for a representative list of cultivars). Despite the fact that *Lr34* is present in few breeding lines and germplasm, this most renowned durable LR resistance gene is still absent in all released SRWW cultivars to date (Kolmer et al., 2018). A complete update on wheat rust across the US including available *Lr* and *Yr* genes and gene postulations on wheat cultivars can be found at the USDA-ARS Cereal Disease Laboratory (https://www.ars.usda.gov/midwest-area/stpaul/cereal-disease-lab/).

There have been few QTL mapping studies for rust resistance conducted in the southeast region. Three biparental QTL mapping studies were recently conducted for LR: one study looked at accessing seedling resistance (Sapkota et al., 2019b) and two focused on APR (**Table 4**) (Carpenter et al., 2017; Sapkota, 2019). The studies utilized RIL population (175–225 lines) and detected major QTL for LR resistance in *2BS* (seedling), *1AL* and *5B* (APR) explaining 8.1–75.3% of phenotypic variations which were either validated or under validation for future use. Among those, *LrA2k* recently mapped on chromosome *2BS* from SRWW cultivar AGS 2000 confers a high level of LR resistance at the seedling stage (Sapkota et al., 2019b). Similarly, *Lr2K38* has been mapped on *1AL* chromosome from SRWW cultivar AGS 2038 and confers a high level of APR to LR (Sapkota et al., 2019). Both of these genes were effective against the current prevalent *Pt* races MFGKG and MBTNB and are invaluable genetic resources for resistance breeding in the southeast region. In addition, a validation study was conducted in the growth chamber for seedling resistance using RILs (**Table 4**) (Carpenter et al., 2018). Linkage analysis validated the presence of *Lr18* gene linked to SNP marker *IWB41960* in Jamestown/VA10W-21 and RL6009/VA10W21 F2 populations which can be successfully deployed in breeding programs.

**Table 4:** Summary of recently identified QTL for LR and SR resistances and their validation in SRWW in the southeast US.

Mapping population	Population size^a^	Number of experiments	Traits studied^b^	Gene/QTL identified/validated	Linked markers	*R^2^*^c^	References
***QTL Identification***							
Pioneer ‘25R47’/Jamestown	186 RILs	Field (6)	IT (0-9 scale), DS	*QLr.vt-5B1* *QLr.vt-5B2*	*IWB7835/IWB24418* *IWB32871/IWB26068*	22.1 8.1	Carpenter et al., 2017
AGS 2038/UGA 111729	225 RILs	Field (3); greenhouse (1)	IT (0-4 scale), DS	*Lr2K38*	*IWB20487*	34.4	Sapkota, 2019
Pioneer® variety 26R61/AGS 2000	175 RILs	Growth chamber (3)	IT (0-4 scale)	*LrA2K*	*Xwmc770*	75.3	Sapkota et al., 2019b
Pioneer variety® 26R61/AGS 2000	178 RILs	Field (3)	IT (0-9 scale)	*YrR61*	*Xbarc124/Xgwm359*	56.0	Hao et al., 2011
VA00W-38/Pioneer Brand 26R46	182 RILS	Field (4)	IT (0-9 scale), DS	*QYrva.vt-2AS* *QYrva.vt-4BL* *QYrpi.vt-6BL*	*Xgwm296b* *Xbarc163* *Xwmc756*	58.9 19.3 11.7	Christopher et al., 2013
VA96W-270/Coker 9835	146 RILs	Field (4)	DS and AUDPC	*QYr.ar-3BS* *QYr.ar-4BL*	*IWA6471* *IWA3551*	28.3 26.3	Subramanian et al., 2016
Pioneer ‘25R47’/Jamestown	186 RILs	Field (6)	IT (0-9 scale), DS	*QYr.vt-3B* *QYr.vt-6A*	*IWB60574/IWB23272* *IWB5971/IWB63000*	11.1 14.3	Carpenter et al., 2017
***QTL Validation***							
Pioneer ‘25R47’/Jamestown	186 RILs; 1600 individuals (200 from each fight F2 population)	Growth chamber (1)	IT (0-4 scale)	*Lr18*	*IWB41960*	–	Carpenter et al., 2018

^a^RILs, recombinant inbred lines.

^b^IT, infection type; DS, disease severity; AUDPC, area under disease progress curve.

^c^The highest amount of phenotypic variation (%) explained by the QTL.

Similarly, four biparental QTL mapping studies were conducted for adult plant SR resistance (**Table 4**) (Hao et al., 2011; Christopher et al., 2013; Subramanian et al., 2016; Carpenter et al., 2017). The first study utilized a RIL population (178 lines) and detected a major QTL on *2A*, contributing up to 56% of phenotypic variation in the SRWW cultivars Pioneer 26R61 (Hao et al., 2011). The second study utilized 182 RILs derived from VA00W-38 (resistant) and Pioneer Brand 26R46 and mapped four QTLs, on chromosomes *2AS, 3BL, 4BL*, and *6BL* (Christopher et al., 2013). Of these, *2AS* QTL explained up to 58.9% of phenotypic variation. The third study utilized a RIL population (146 RILs) and mapped two QTLs on chromosomes *3BS* and *4BL* (Subramanian et al., 2016). The fourth study utilized a RIL population (186 lines) and detected two QTLs for SR resistance in SRWW cultivar Jamestown in chromosomes *3B* and *6A*, contributing up to 11.1 and 14.3% of phenotypic variation, respectively (Carpenter et al., 2017). Interestingly, the presence of LR and SR resistance QTL on *5B, 3B*, and *6A* together with FHB resistance QTL in *1B* makes SRWW cultivar Jamestown a good donor parent in the SRWW breeding programs (Carpenter et al., 2017). In addition, donor parents Pioneer26R61 and VA00W-38 in these mapping studies acquiring FHB and LR resistance QTL besides SR resistance hold a good genetic pool for multi-trait resistance.

## Challenges and Breeding Strategies for FHB, LR, and SR Resistance

Disease phenotyping in the greenhouse and in particular under field conditions is necessary to validate germplasm selections based on molecular data. However, FHB and rusts differ dramatically on the relative ease of disease phenotyping. Disease ratings for rust are fairly straightforward and consider infection type (either a 0–4 or 0–9 scale) and disease severity to assess both seedling and APR (**Figure 2**) (Stakman et al., 1962; Qayoum and Line, 1985; Carpenter et al., 2017). Inoculum can be either urediniospore suspensions (greenhouse) or infected spreader plants (field) to initiate localized epidemics. The presence of five resistance types and the need for floral infection for FHB assessments increase the difficulty of phenotyping for this disease (**Figures 1** and **3**) (Mesterhazy, 1995). Field and greenhouse assessments typically require an exogenous water source (*e.g.* mist system) that can be programmed to provide moisture throughout the day and night during anthesis. This can often be over several week periods due to differing anthesis across heterogeneous germplasm. Inoculum can be applied as corn spawn and conidial spray inoculation or naturally infected corn debris in the field to assess all resistance types while single floret inoculations of a conidial suspension are typically used in the greenhouse particularly to assess type II, III, and IV resistance. Differences in plant height and flowering date of wheat germplasm may require multiple inoculations creating uneven disease pressure in the field with the likelihood of disease escape on early and later flowering germplasm. High disease incidence resulting in a majority of infected spikelets can also make type II screening challenging (M. Mergoum, *personal communication*). Additionally, *Stagonospora nodorum* infection on the wheat spike may add confusion to correctly rate FHB in the field (Brown-Guedira et al., 2008).

The selection of wheat cultivars with reduced FDK and DON levels is becoming a major criterion in the majority of the southeast wheat breeding programs. However, collecting these data requires intensive labor and resources for large and early generation germplasm selections. Several research groups are developing high throughput phenotyping such as detached leaf assays (Browne et al., 2005), air separation and digital photo analysis (Maloney et al., 2014), near-infrared reflectance (Dvorjak, 2014), optical sorter (Carmack et al., 2019) or grain imaging platform (Ward et al., 2019a) and have observed a high level of correlation for FDK and DON level between actual and predicted values. Despite these achievements, there are still some other impediments to successful field phenotyping for both FHB and rust diseases.

Low heritability and high genotype by environment interaction (G×E) are the major bottlenecks to the complexity of FHB resistance breeding as it is usually challenging to detect minor effect QTL both from adapted and exotic sources (Verges et al., 2006). Plant breeders can have hundreds of thousands of breeding lines per season and are limited in the greenhouse for FHB and rust screening due to time and space constraints. High G×E can result in a poor correlation between greenhouse and field screening for quantitative traits such as FHB severity, DON levels, and FDK which suggests that greenhouse test alone would not be sufficient to explore resistance sources which might actually be expressed only in the field (Hall and Van Sanford, 2003). Regardless of the time and effort, phenotypic screening in multiple environments is still indispensable to lay the foundation for successful MAS and GS. A recent study showed that the incorporation of the G×E effect and multiple traits in the GS model increased the prediction accuracy by 9.6% for low-heritability traits across environments (Ward et al., 2019b). Notably, the higher prediction ability of the phenotypic selection over GS with higher selection response *vice versa* documented by Steiner et al. (2019) underscores that genomics and phenomics should be complementary to further cultivar improvements. Similarly, the higher accuracy of genome-wide maker-based models tested in recent studies to detect even minor effect QTL unchecked by MAS ensures the success of GS for future rusts resistance in SRWW (Todorovska et al., 2009; Bulli et al., 2016; Steiner et al., 2017).

Though significant strides have been made in FHB resistance using universal donor Sumai 3, inadvertent linkage drag associated with *Fhb1* has been a problem for its successful deployment in breeding lines (Schweiger et al., 2016). It is often challenging to gain acceptable agronomic and quality traits while introgressing major effect FHB resistance QTL from exotic and alien sources. Benson et al. (2012) studied the genetic diversity and population structure of the eastern winter wheat population and concluded that the short intense breeding history against FHB resistance might have localized effects on linkage drag. However, appropriate breeding methodologies such as repeated backcrossing with the recurrent parent and large population size can help to identify the few recombinants with minimum negative effects (“bad linkages”). More importantly, breeding winter wheat for both FHB and rusts resistance is time consuming (requires vernalization) compared to spring wheat. In addition, the lack of QTL validation in diverse backgrounds and the paucity of diagnostic markers have disadvantaged FHB and rusts breeding programs in the southeast US (Brown-Guedira et al., 2008; Waqar et al., 2018). Low accuracy in QTL size and location along with a low representation of genomic allele distribution often warrants the need for validating QTL and associated markers in a diverse background to ensure wider applicability (Buerstmayr et al., 2019).

Unlike FHB, the emergence of new virulence phenotype groups and races of the LR and SR pathogens is the single most recurring problem for durable rust resistance breeding in the southeast US and elsewhere (Kolmer et al., 2019). The replacement of LR virulence phenotype MBRK and TLGF dominant during the early 2000s with MBTNB and MCTNB phenotypes at present highlights the dynamic nature of LR isolates (Kolmer, 2002; Kolmer et al., 2019). Similarly, new races of SR have been continuously appearing since races virulent to *Yr8* and *Yr9* started threatening SRWW in the southeast US in 2000 (Hao et al., 2011). Wheat breeding programs in the southeast US should, therefore, continue the rigorous search for novel QTL and slow rusting genes for LR and SR resistance to overcome new races and gain long term genetic resistance in the field. In addition, stewardship and deployment of the few resistance genes for LR and SR available to breeding programs in the southeast are necessary to preserve and prolong the efficacy of these genes.

With the recent achievements on whole-genome sequencing (IWGSC, 2014) and the genome annotation (Appels et al., 2018), the future breeding endeavor of SRWW should orient towards the exploration of high-throughput genomic tools such as field pathogenomics, transgenes, gene cloning, genome editing, NGS, and GBS to gain deeper insights into host and underlying pathogen to achieve durable FHB and rust resistance (Bulli et al., 2016; Dinh et al., 2020; Figlan et al., 2020). Above all, a continued collaboration among wheat breeders for sharing and multi-location screening of the promising germplasm against FHB, LR, and SR resistance will be a key to successful resistance breeding in the southeast US.

## Author Contributions

BG, SS, and BB respectively wrote the major sections on FHB, LR, and SR of the manuscript. BG designed the overall outline and prepared the first draft of the manuscript. JB, MM, and AM-E provided guidance, critical suggestions, and feedback on the overall content throughout the manuscript preparation. MM initiated the concept of this chapter and led the group in accomplishing it efficiently and timely. All authors contributed to the article and approved the submitted version.

## Conflict of Interest

The authors declare that the research was conducted in the absence of any commercial or financial relationships that could be construed as a potential conflict of interest.
